# Burden of Childhood Diarrhea and Its Associated Factors in Ethiopia: A Review of Observational Studies

**DOI:** 10.3389/ijph.2024.1606399

**Published:** 2024-06-05

**Authors:** Biniyam Sahiledengle, Daniel Atlaw, Lillian Mwanri, Pammla Petrucka, Abera Kumie, Yohannes Tekalegn, Fikreab Desta, Demisu Zenbaba, Telila Mesfin, Degefa Gomora, Kingsley Emwinyore Agho

**Affiliations:** ^1^ Department of Public Health, Madda Walabu University Goba Referral Hospital, Bale-Goba, Ethiopia; ^2^ Department of Human Anatomy, Madda Walabu University Goba Referral Hospital, Bale-Goba, Ethiopia; ^3^ Research Centre for Public Health, Equity and Human Flourishing, Torrens University Australia, Adelaide Campus, Adelaide, SA, Australia; ^4^ College of Nursing, University of Saskatchewan, Saskatoon, SK, Canada; ^5^ School of Public Health, College of Health Science, Addis Ababa University, Addis Ababa, Ethiopia; ^6^ School of Medicine, Madda Walabu University Goba Referral Hospital, Bale-Goba, Ethiopia; ^7^ Department of Midwifery, Madda Walabu University Goba Referral Hospital, Bale-Goba, Ethiopia; ^8^ School of Health Sciences, Western Sydney University, Penrith, NSW, Australia

**Keywords:** diarrhea, Ethiopia, risk factors, under-five children, WASH

## Abstract

**Objectives:** This systematic review and meta-analysis aimed to: i) determine the pooled prevalence of acute diarrhea; and ii) synthesize and summarize current evidence on factors of acute diarrheal illnesses among under-five children in Ethiopia.

**Methods:** A comprehensive systematic search was conducted in PubMed, SCOPUS, HINARI, Science Direct, Google Scholar, Global Index Medicus, Directory of Open Access Journals (DOAJ), and the Cochrane Library. This systematic review and meta-analysis followed the Preferred Reporting Items for Systematic Reviews and Meta-Analyses (PRISMA) guideline. The methodological quality of each included article was assessed using the Joanna Briggs Institute (JBI) quality assessment tool for cross-sectional and case-control studies. A random-effect meta-analysis model was used to estimate the pooled prevalence of diarrheal illnesses. Heterogeneity and publication bias were assessed using I^2^ test statistics and Egger’s test, respectively. The statistical analysis was done using STATA™ software version 14.

**Results:** Fifty-three studies covering over 27,458 under-five children who met the inclusion criteria were included. The pooled prevalence of diarrhea among under-five children in Ethiopia was found to be 20.8% (95% CI: 18.69–22.84, n = 44, I^2^ = 94.9%, *p* < 0.001). Our analysis revealed a higher prevalence of childhood diarrhea in age groups of 12–23 months 25.42% (95%CI: 21.50–29.35, I^2^ = 89.4%, *p* < 0.001). In general, the evidence suggests that diarrheal risk factors could include: i) child level determinants (child’s age 0–23 months, not being vaccinated against rotavirus, lack of exclusive breastfeeding, and being an under-nourished child); ii) parental level determinants {mothers poor handwashing practices [pooled odds ratio (OR) = 3.05; 95% CI:2.08–4.54] and a history of maternal recent diarrhea (pooled OR = 3.19, 95%CI: 1.94–5.25)}; and iii) Water, Sanitation and Hygiene (WASH) determinants [lack of toilet facility (pooled OR = 1.56, 95%CI: 1.05–2.33)], lack handwashing facility (pooled OR = 4.16, 95%CI: 2.49–6.95) and not treating drinking water (pooled OR = 2.28, 95% CI: 1.50–3.46).

**Conclusion:** In Ethiopia, the prevalence of diarrhea among children under the age of five remains high and is still a public health problem. The contributing factors to acute diarrheal illnesses were child, parental, and WASH factors. A continued focus on improving access to WASH facilities, along with enhancing maternal hygiene behavior will accelerate reductions in diarrheal disease burden in Ethiopia.

## Introduction

Childhood diarrheal disease remains to be the existential threat to global public health scourge. According to the Global Burden of Disease Study 2019, diarrhea disease was listed among the top three most common problems causing a significant health burden in children [1]. Evidence indicates that deaths from diarrheal disease among children under 5 are most prevalent in South Asia and sub-Saharan Africa, where access to healthcare, safe water, and sanitation remains limited [2, 3]. In Ethiopia, although there has been a reduction in the prevalence of diarrheal disease from 24% in 2000 to 12% in 2016, progress has not been sufficiently rapid to fully tackle this significant public health issue [4, 5].

Previous studies conducted in Ethiopia have identified many factors that are associated with childhood diarrhea. These factors include: child’s age [6–10], place of residency [11–13], lack of exclusive breastfeeding [14–17], unvaccinated against rotavirus [7, 14, 18–22], undernutrition [23–25], limited maternal education [11, 23, 24, 26], inadequate knowledge about diarrheal disease [8, 27], poor handwashing practices [15, 24, 28, 29], low wealth status [14, 26, 27, 30, 31], unimproved sources of drinking water [6, 9, 12, 13, 19, 32], and unimproved toilet facilities [7, 19, 20, 32–36].

Despite the fact that diarrhea is largely preventable, it remains a public health problem in Ethiopia, and its burden is still a serious concern. Estimates of the burden of diarrheal disease and its associated factors in Ethiopia at the national level are not well known, masking the current status, a prohibitive factor to tracking progress and reducing morbidity. While it is known this to be an ongoing and significant public health issue needing current evidence, the most review evidence have been over 5 years old [37], which may not reflect the present situation. Additionally, preliminary studies that have recently been conducted throughout the country have reported inconsistent findings [7, 12, 13, 26, 31, 33, 34, 38–44], making it necessary to update the existing review in order to estimate the current nationwide and regional pooled prevalence of diarrheal diseases and associated factors. Therefore, this systematic review and meta-analysis provide updated results of pooled estimates of childhood diarrhea morbidity and summarized its associated factors in Ethiopia. The findings affirm the current status of diarrheal diseases and set a benchmark for tracking the progress toward achieving the Sustainable Development Goals (SDG).

## Methods

### Protocol Registry

The protocol for this review was registered in the International Prospective Register of Systematic Reviews (PROSPERO), the University of York Centre for Reviews and Dissemination (record ID: CRD42022354416).

### Search Strategy and Information Sources

This is an update of the systematic review and meta-analysis, which was conducted using the Preferred Reporting Items for Systematic Reviews and Meta-Analysis (PRISMA) guidelines 2020 [45] (Supplementary Material S1).

### Eligibility Criteria

*Population*: children under the age of 5 years (under-fives).

*The outcome of interest*: prevalence of acute diarrheal illness. Studies that reported diarrhea above 2 weeks were excluded.

*Study design*: Observational studies (cohort, case-control, and cross-sectional studies) that assessed the prevalence and associated factors of under-five diarrheal disease were included in the study. Any observational studies that did not report either the prevalence or associated factors of acute diarrhea by controlling possible confounders were excluded from the study. Additionally, interventional studies (randomized controlled trials (RCTs), cluster randomized controlled trials (cRCTs) and quasi-experimental (QE) trials), systematic reviews, commentaries, letters to editors, qualitative studies, case studies, books, reports, conference abstracts, non-primary studies, and analysis of policy briefs were excluded.

*Study setting:* Only studies conducted in Ethiopia.

*Publication status*: Both published and unpublished studies were considered.

*Language*: Articles published in the English language were considered.

*Publication year*: All articles published since 2017 were considered.

### Operational Definition

Acute diarrhea: is defined as the passage of three or more loose or liquid stools per day or an increase in stool frequency or liquidity that is considered abnormal during the 2 weeks as reported by the mothers or caregivers [46].

### Information Sources

A comprehensive review of English language literature using list of relevant Medical Subject Heading Terms (MeSH) words and sub-headings of keywords was generated and used to search articles from major databases including PubMed, SCOPUS, HINARY, Science Direct, Google Scholar, Global Index Medicus, Directory of Open Access Journals (DOAJ), and the Cochrane Library. Each database’s or search engine’s retrieved articles were added to an EndNote library. Additionally, articles from the retrieved bibliographies that met the inclusion criteria were added to be reviewed. We also used Google Scholar and Google to track citations.

### Searching Strategy

The following combination of keywords and other MeSH words was used in the search: diarrhea [MeSH Terms], Diarrhea [Text Word], Diarrhoea [All Filed], Prevalence [Text Word], Factor*, determinant*, child [MeSH Terms], infant [MeSH Terms], under-five children, and Ethiopia. The search terms were used separately and in combination using Boolean operators like “OR” or “AND.” A detail searching strategy was provide in the Supplementary Material S2. The electronic database search was supplemented with gray literature searches via Google Scholar and Google searches. A secondary search method known as “footnote chasing” was also used to identify further and relevant articles. Reference lists of included studies were manually searched for additional relevant articles. Moreover, preprint articles from the MedRxiv, BioRxiv, and Research square databases were also accessed to ensure wider coverage. The related systematic review and meta-analysis published in 2017, in which the subjects from the included studies were mostly enrolled before 2017. In this review, all papers published from first of November 2017 to June 30th^,^ 2022, were considered. All papers published until the June 30th, 2022 were considered. An initial search was conducted on June 1st^,^ 2022 and later repeated on June 30th, 2022 to identify articles.

### Study Selection Process

In accordance with predetermined inclusion and exclusion criteria, two investigators (BS and DA) independently screened and identified relevant articles by title, abstract, and full text. The screened articles were compiled by the two authors (BS and DA), and any disagreements between them were settled through discussion. Duplicate articles were then removed from the review after all the searched articles were exported into the EndNote™ version X8 software. After reading the complete texts of the remaining articles, we retained studies that met the inclusion criteria. Based on the eligibility requirements, BS and DA independently reviewed the full text of the articles based on the study eligibility criteria.

### Data Extraction

Microsoft™ Excel was used to extract the data. The primary author, publication year, study design, study area, sample size, prevalence of diarrhea, number of children, age of children, sampling techniques, and factors associated with diarrhea were included in the summary of the studies that were included in the review.

### Risk of Bias Assessment of the Studies

The quality of the included studies was evaluated using the Joanna Briggs Institute (JBI) method for quality evaluation. We used the JBI checklist for case-control studies and observational studies were used [47]. Two reviewers independently evaluated the included studies’ quality (BS and DA). There are eight and ten parameters in the evaluation tools for cross-sectional and case-control studies, respectively. When the information provided was insufficient to make a decision, we decided to assess a 1 rating for the specific item (a failure to satisfy a specific item or unclear/not applicable). Bias risks were divided into three categories: low (0–2), moderate (3 or 4), and high (total score of 5 or higher). Disagreements are usually remedied through discussion until consensus is reached. However, for the current study, there was no disagreement in selecting the potential research (Supplementary Material S3).

### Synthesis of Results

The prevalence of diarrhea and/or associated factors were used in reporting the findings of each study. Where available, 95% CI for reported diarrhea was obtained from the eligible studies. Based on published studies, 95% CI were calculated using available data where the full text of the eligible studies did not report diarrheal estimates. Narrative methods including text and table were used as a tool for associated factors data presentation. The heterogeneity among included studies was assessed by I^2^ statistics and the Cochran Q-test. The included studies exhibited significant high heterogeneity (I^2^ = 94.9%, *p* < 0.001), which led us to compute a random effect meta-analysis model to estimate the pooled prevalence of diarrhea. The pooled prevalence of diarrhea and their corresponding 95% CI were presented using a forest plot. Subgroup analyses were performed to investigate the observed heterogeneity, based on the sub-regions of Ethiopia. Further statistical analyses, such as univariate meta-regression, was also performed to identify possible sources of heterogeneity.

We used a qualitative approach to summarize the factors associated with diarrhea and synthesize the relevant information based on the objectives of the study. We did not perform a meta-analysis for all identified factors due to the considerable heterogeneity of the included studies. We have discussed identified associated factors and estimates descriptively. For some variables, however, the adjusted odds ratios (AOR) were pooled using the generic inverse variance method, which involved converting the adjusted odds ratio to a logarithmic scale and then calculating standard error based on the 95% confidence intervals [48]. The Cochran Q-test and Haggin I^2^ statistics were used to assess the presence and degree of heterogeneity among included studies [49].

### Publication Bias

In this meta-analysis, the presence of publication bias was evaluated using funnel plots and Egger’s weighted regression test at a significance level of less than 0.05.

### Sensitivity Analysis

To identify the source of heterogeneity, a leave-one-out analysis was employed. Sensitivity analysis using a random-effects model was performed to assess the influence of a single study on the overall pooled prevalence estimate.

## Results

### Study Selection

**FIGURE 1:**
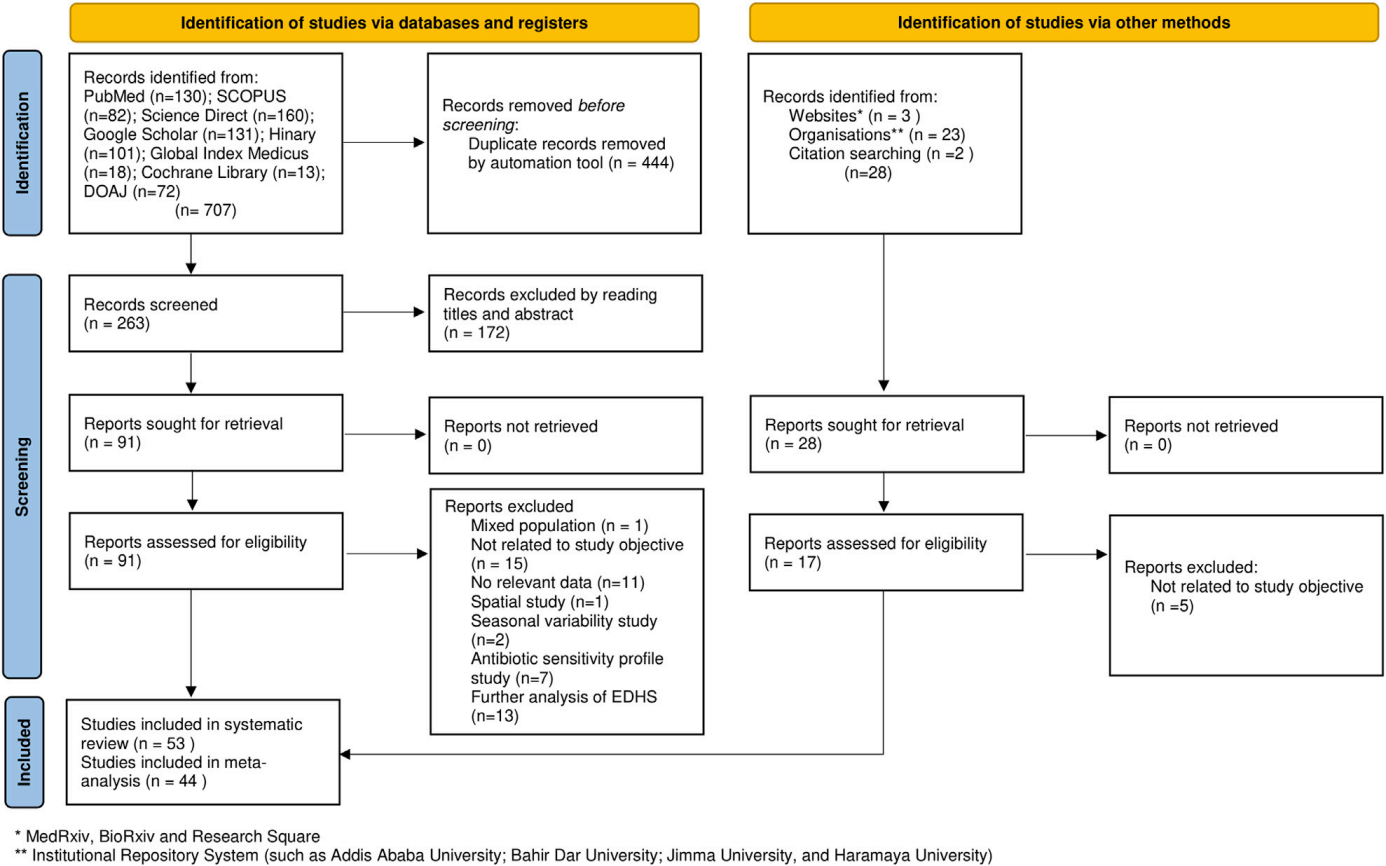
Flow chart of study selection for systematic review and meta-analysis of prevalence of acute diarrhea and associated factors among under-five children in Ethiopia, (Ethiopia, 2017–2022).

Overall, the searches identified 707 articles (i.e., identification of studies via databases and registers). Of the initial articles, 444 articles were excluded due to duplication. We screened the titles and abstracts of 263 articles and obtained 91 full text articles, of which 53 studies met the inclusion criteria and were included in the final systematic review and meta-analysis. Of the included 53 studies, 44 studies were eligible for meta-analysis (Figure 1).

### Characteristics of Included Studies

The descriptive summary of included cross-sectional and case-control studies were presented in Table 1. Of the included 53 studies, 9 were case-control studies, 12 comparative cross-sectional studies, and 32 cross-sectional studies by design. A total of 27,458 under-five children were included as participants in the current systematic review and meta-analysis. In the current review, the sample size varied from small (n = 191) [61] to large (n = 2,030) [6]. In this review, the lowest prevalence of diarrhea (9.4%) was found in a study conducted in Bahir Dar, northwest Ethiopia Amhara region [14], while the highest prevalence (36.5%) of diarrhea was reported in a study conducted at west Guji zone, Oromia region [62].

**TABLE 1:** Descriptive summary of studies included in this systematic review and meta-analysis of the prevalence and risk factors of diarrhea among under-five children in Ethiopia (Ethiopia, 2017–2022).

	Author	Publication year	Sample size	Prevalence, 95%CI	Study design	Study population	Region	Sampling method
1	Alemayehu M et al. [19]	2020	722	23.5 (20.4–26.6)	CS	0–59 months	SNNPR	Multistage sampling technique
2	Hailu B et al. [33]	2021	419	25.3 (21.1–29.5)	CS	0–4 years	Amhara	Systematic sampling technique
3	Tesfaye TS et al. [34]	2020	311	30.9 (25.8–36.0)	CS	0–59 months	SNNPR	Systematic sampling technique
4	Soboksa NE et al. [50]	2021	756	19.8 (17.0–22.6)	CCS	0–59 months	Oromia	Multistage sampling technique
5	Getahun W et al. [41]	2021	484	17.6 (14.2–21.0)	CS	0–59 months	Amhara	Systematic sampling technique
6	Dagnew AB et al. [38]	2019	498	14.5 (11.4–17.6)	CS	0–59 months	Amhara	Systematic sampling technique
7	Getachew A et al. [51]	2018	736	22.1 (19.1–25.1)	CS	0–59 months	Amhara	Systematic sampling technique
8	Gebrezgiabher BB et al. [35]	2019	652	13.9 (11.2–16.6)	CCS	0–59 months	Tigray	Multistage sampling technique
9	Wasihun AG et al. [42]	2018	610	27.2 (23.7–30.7)	CS	6–59 months	Tigray	Multistage stratified sampling
10	Ayalew AM et al. [52]	2018	525	23.0 (19.4–26.6)	CCS	0–59 months	Amhara	Multistage sampling technique
11	Adane M et al. [30]	2018	690	11.9 (9.5–14.3)	CS	0–50 months	Addis Ababa	Multistage sampling technique
12	Melese B et al. [24]	2019	537	13.6 (10.7–16.5)	CS	0–59 months	Sidama	Multistage sampling technique
13	Shumetie G et al. [14]	2018	553	9.4 (7.0–11.8)	CS	0–59 months	Amhara	Multistage sampling technique
14	Mekonnen GK et al. [36]	2019	813	33 (29.8–36.2)	CCS	0–59 months	Gambella	Stratified multistage sampling
15	Degebasa MZ et al. [53]	2018	760	25 (21.9–28.1)	CCS	0–59 months	Addis Ababa	Multistage sampling method
16	Mernie G et al. [40]	2022	654	15.7 (12.9–18.5)	CCS	0–59 months	Amhara	Multistage sampling technique
17	Wagari S et al. [54]	2022	535	24.8 (21.1–28.5)	CS	0–59 months	Oromia	Simple random sampling technique
18	Feleke Y et al. [15]	2022	440	17.3 (13.8–20.8)	CS	0–59 months	Oromia	Systematic sampling technique
19	Fufa WK et al. [55]	2019	559	22 (18.6–25.4)	CCS	6–59 months	Oromia	Multistage cluster sampling
20	Feleke DG et al. [56]	2022	717	14.5 (11.9–17.1)	CS	0–59 months	Amhara	Two stage multi-stage sampling
21	Megersa S et al. [57]	2019	709	20.2 (17.2–23.2)	CCS	0–59 months	Oromia	Two stage multi-stage sampling
22	Beyene SG et al. [58]	2018	450	28.4 (24.2–32.6)	CS	0–59 months	Oromia	Multistage sampling
23	Kasee LF et al. [59]	2018	250	14.9 (10.5–19.3)	CS	0–59 months	Oromia	Systematic sampling
24	Gashaw TA et al. [23]	2019	582	21.8 (18.4–25.2)	CS	0–59 months	SNNPR	Multi-stage sampling
25	Bitew BD et al. [31]	2022	407	24.9 (20.7–29.1)	CS	0–59 months	Amhara	Simple random sampling technique
26	Getachew F et al. [60]	2022	450	13.6 (10.4–16.8)	CS	0–59 months	Addis Ababa	Systematic sampling technique
27	Zedie FB et al. [28]	2018	808	14.7 (12.3–17.1)	CCS	0–59 months	Sidama	Multistage sampling technique
28	Chomissa AR et al. [20]	2018	718	16.7 (14.0–19.4)	CCS	0–59 months	SNNPR	Multistage sampling technique
29	Arba A et al. [89]	2020	223	27.3 (21.5–33.1)	CS	0–59 months	SNNPR	Systematic sampling
30	Amamo DD et al. [62]	2019	717	36.5 (33.0–40.0)	CS	0–59 months	Oromia	Multistage cluster random sampling
31	Zegeye Z [90]	2021	554	26.4 (22.7–30.1)	CS	0–59 months	Amhara	Multistage sampling technique
32	Mengistu KD [61]	2021	191	25.7 (19.5–31.9)	CS	0–59 months	Amhara	Simple random sampling technique
33	Angasu K et al. [6]	2022	2030	34.5 (32.4–36.6)	CS	0–59 months	SNNPR	Two-stage sampling procedure
34	Mitiku HD [13]	2021	873	19.8 (17.2–22.4)	CS	0–59 months	Amhara	Systematic sampling technique
35	Kassie G [91]	2020	661	11 (8.6–13.4)	CS	2–59 months	Amhara	Multistage sampling
36	Alemayehu B et al. [21]	2020	826	18.3 (15.7–20.9)	CS	0–59 months	SNNPR	Stratified sampling
37	Natnael T et al. [9]	2021	335	11 (7.7–14.3)	CS	0–59 months	Amhara	Systematic sampling
38	Bekele D et al. [22]	2021	512	17.8 (14.5–21.1)	CCS	0–59 months	Oromia	Multi-stage sampling technique
39	Fenta A et al. [43]	2020	717	14.5 (11.9–17.1)	CS	0–59 months	Beni Shangul Gumuz	Multistage sampling
40	Alemayehu K et al. [8]	2021	620	24 (20.6–27.4)	CS	0–59 months	Oromia	Simple random sampling
41	Shine S et al. [18]	2020	420	16.4 (12.9–19.9)	CS	0–59 months	Amhara	Multi-stage sampling
42	Solomon ET et al. [44]	2020	1,146	23 (20.6–25.4)	CS	0–59 months	Dire Dawa	Multi-stage sampling procedure
43	Tafere Y et al. [26]	2020	758	29.9 (26.6–33.2)	CCS	0–59 months	Amhara	Systematic sampling technique
44	Mulu E et al. [32]	2022	530	21.3 (17.8–24.8)	CS	0–59 months	SNNPR	Multi-stage sampling procedures
45	Brhanu H et al. [11]	2017	618	-	CC	0–59 months	Benishangul Gumuz	Simple random sampling technique
46	Derseh BT et al. [27]	2021	309	-	CC	6–59 months	Amhara	Systematic sampling technique
47	Delelegn MW et al. [39]	2020	306	-	CC	0–59 months	Amhara	Systematic sampling technique
48	Mosisa D et al. [7]	2021	399	-	CC	0–59 months	Oromia	Systematic sampling technique
49	Baye A et al. [16]	2021	357	-	CC	0–23 months	Amhara	Simple random sampling technique
50	Soboksa NE et al. [29]	2020	396	-	CC	0–59 months	Oromia	Simple random sampling technique
51	Girma M et al. [12]	2018	469	-	CC	0–59 months	Amhara	Multi-stage sampling procedures
52	Brhanemeskel H [63]	2021	155	-	CC	6–23 months	Addis Ababa	Simple random sampling technique
53	Getachew B et al. [17]	2018	352	-	CC	0–59 months	Harari	Simple random sampling technique

CS: cross-sectional study; CCS: comparative cross-sectional study; CC: case-control study.

### Meta-Analysis

**FIGURE 2:**
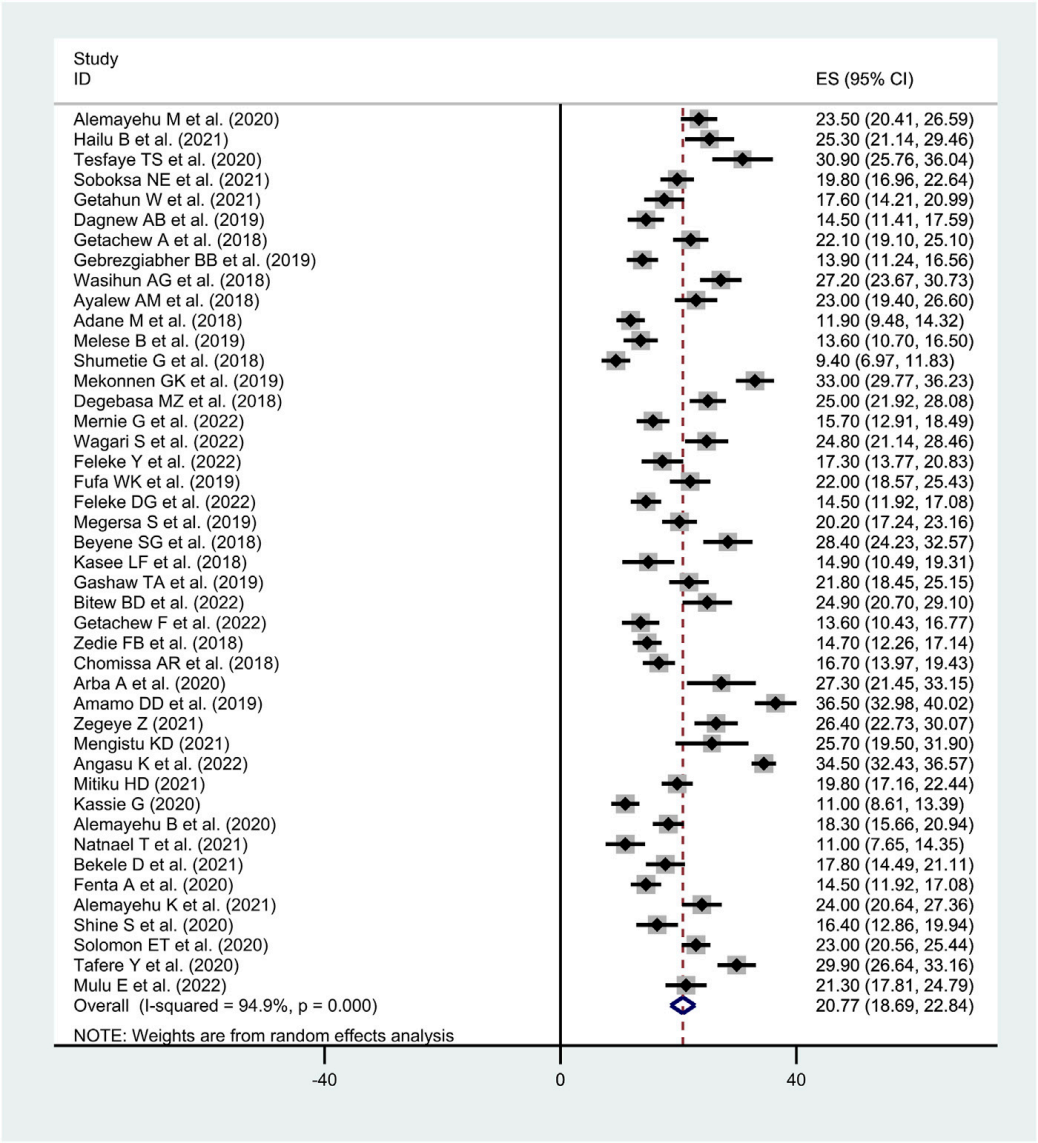
Forest plot of the pooled prevalence of diarrhea among under-five children in Ethiopia, (Ethiopia, 2017–2022).

The meta-analysis included only studies with a cross-sectional design. The overall pooled prevalence of diarrhea among under-five children in Ethiopia was found to be 20.8% (95% CI: 18.69–22.84, I^2^ = 94.9%, *p* < 0.001) (Figure 2).

We performed subgroup analysis based the studies’ geographical settings (i.e., region of the country). Accordingly, the highest proportion of acute diarrhea was observed in Gambella region with a prevalence of 33.0% (95% CI: 29.76–36.23) followed by SNNPR region with a prevalence of 24.2% (95% CI: 18.94–29.47), Dire Dawa city administration 23.0% (95% CI: 20.56–25.44), Oromia 22.6% (95% CI: 18.85–26.28), Amhara region 19.1% (95% CI: 15.95–22.14) and the lowest proportion was reported from Sidama region at 14.2% (95% CI: 12.37–16.11) (Table 2).

**TABLE 2:** Subgroup analysis of acute diarrhea among under-five children in Ethiopia (Ethiopia, 2017–2022).

Sub-group	Number of studies (n)	Prevalence of diarrhea	95%CI	I^2^ (%)	*p*-value
Sex					
Male	16	19.73	15.39–24.07	94.1	*p* < 0.001
Female	16	19.74	15.70–23.78	93.1	*p* < 0.001
Age of the child					
0–5 months	15	16.14	9.65–22.63	92.5	*p* < 0.001
6–11 months	19	25.34	21.36–29.32	83.0	*p* < 0.001
12–23 months	22	25.42	21.50–29.35	89.4	*p* < 0.001
24–59 months	20	19.56	15.37–23.75	94.9	*p* < 0.001
Regions					
Amhara	16	19.05	15.95–22.14	93.4	*p* < 0.001
Oromia	10	22.56	18.85–26.28	91.3	*p* < 0.001
SNNPR	8	24.21	18.94–29.47	95.5	*p* < 0.001
Addis Ababa	3	16.80	8.84–24.77	95.7	*p* < 0.001
Tigray	2	20.49	7.46–33.53	97.1	*p* < 0.001
Sidama	2	14.24	12.37–16.11	0.0	0.569
Gambella	1	33.00	29.76–36.23	0.0	-
Beni Shangul Gumuz	1	14.50	11.92–17.08	0.0	-
Dire Dawa	1	23.00	20.56–25.44	0.0	-

We conducted a stratification analysis on prevalence, considering age groups and gender. Our findings revealed that the prevalence of diarrhea among male under-five children in Ethiopia was 19.73% (95% CI: 15.39–24.07, I^2^ = 94.1%, *p* < 0.001), a rate comparable to that of female children. Additionally, our analysis revealed that the prevalence of childhood diarrhea varied across different age groups:0–5 months was 16.14% (95%CI: 9.65–22.63, I^2^ = 92.5%, *p* < 0.001), 6–11 months 25.34% (95%CI: 21.36–29.32, I^2^ = 83.0%, *p* < 0.001), 12–23 months 25.42% (95%CI: 21.50–29.35, I^2^ = 89.4%, *p* < 0.001), and 24–59 months 19.56% (95%CI: 15.37–23.75, I^2^ = 94.9%, *p* < 0.001) (Table 2 and Supplementary Material S4A–D).

### Publication Bias

**FIGURE 3:**
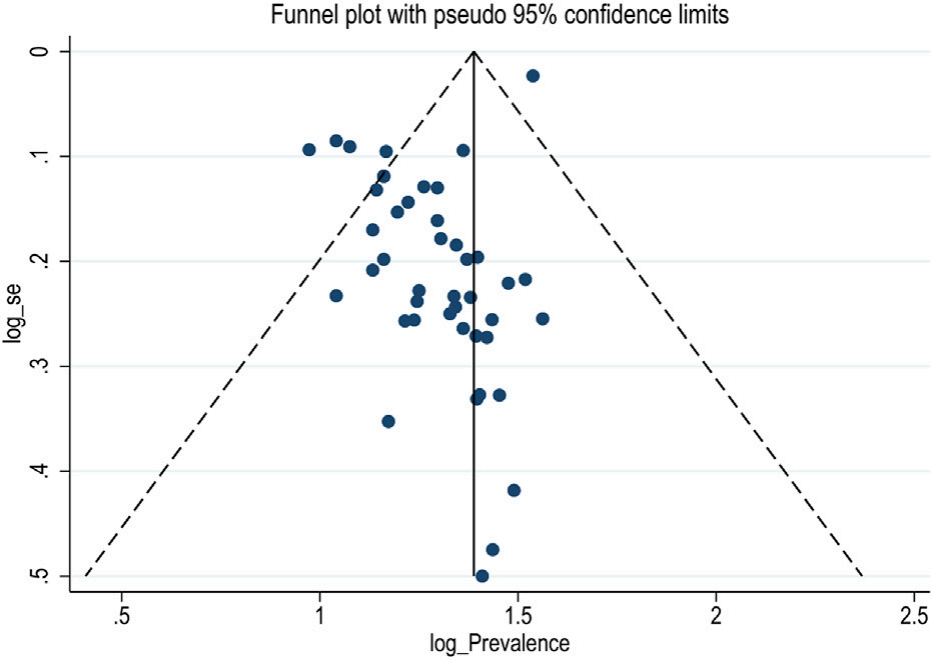
Funnel plot showing publication bias of prevalence of diarrhea among under-five children in Ethiopia, (Ethiopia, 2017–2022).

In this meta-analysis, possible publication bias was visualized through funnel plots and using Egger’s weighted regression test. Asymmetrical large, inverted funnel resembled the absence of publication biases (Figure 3). The Egger’s tests were also not statistically significant for the estimated prevalence of diarrhea in Ethiopia, with a *p*-value of 0.06.

### Sensitivity Analyses

To detect the influence of one study on the overall meta-analysis estimate, sensitivity analysis was conducted using a random-effects model. There was no evidence for the influence of a single study on the overall pooled result of diarrheal morbidity in Ethiopia (Supplementary Material S5).

### Meta-Regression

We conducted a univariate meta-regression analysis by considering publication year, sample size, and quality score as covariates to identify the possible sources of heterogeneity across primary studies, but none of these variables was found to be a statistically significant source of heterogeneity. The results of meta-regression analysis also showed no significant relationship between prevalence of diarrheal disease and sampled size (*p* = 0.084) and publication year (*p* = 0.729).

### Meta-Analysis of Selected Associated Factors and Evidence From the Reviewed Studies

Table 3 demonstrates the summary of the studies included in this review. The factors associated with acute diarrheal disease have been divided into categories of child, parental, and WASH and environmental factors.

**TABLE 3:** Summary of risk factors and there strength of association of acute diarrhea among under-five children in Ethiopia, (Ethiopia, 2017–2022).

Author	Year	Study design	Region	Risk factors and there strength of association		
				Child factors	Parental/household level factors	WASH and environmental factors
Brhanu H et al. [11]	2017	Unmatched case-control	Benishangul Gumuz	• Child’s age (6–11 months) [AOR = 3.85, 95%CI (1.67, 8.86)] • Children from rural areas [AOR = 6.84, 95%CI = (3.26, 14.33)] • Bottle feeding [AOR = 10, 95% CI = (4.56, 21.94)] • Being wasted child [AOR = 2.25, 95%CI = (1.30, 3.91)]	• No formal education [AOR = 4.13, 95% CI (1.54, 11.06)] • Mothers with a history of diarrhea [AOR = 8.81, 95%CI = (4.04, 19.20)]	• Children did not use treated drinking water at home [AOR = 2.46, 95%CI (1.32, 4.57)] • Unsafe child stool disposal [AOR = 2.72, 95%CI = (1.54, 4.81)]
Derseh BT et al. [27]	2021	Unmatched Case-control	Amhara		• Hand washing without soap [AOR = 3.75, 95% CI: (1.16–12.13) • Average family monthly income [AOR = 6.22; 95% CI: 1.30, 29.64)] • Poor knowledge about acute diarrhea (AOR = 15.3; 95% CI: 4.18, 55.88) • Families who consume leftover food (AOR = 5.52; 95% CI: 1.60, 19.03)	• Families who did not treat their drinking water at home (AOR = 9.36; 95% CI: 2.73, 32.08) • Families who dispose infant feces outside the latrine (AOR = 11.01; 95% CI: 3.37, 35.96)
Delelegn MW et al. [39]	2020	Unmatched Case-Control	Amhara	• Children who did not exclusively breastfeed [AOR = 3.32; 95% CI (1.206, 9.14)] • Children who consumed left-over food [AOR = 2.96; 95% CI (1.19, 7.32)]	• Mothers/caregivers did not wash their hands at the critical time [AOR = 5.47; 95% CI:1.68, 17.8] • Mothers had not been counseled by health professionals [AOR = 3.23; 95% CI (1.15, 9.09)] • Mother who had a history of diarrhea in previous 2 weeks [AOR = 6.06; 95% CI (2.42, 15.22)]	• Unsafe child stool disposal [AOR = 4.12; 95% CI (1.25, 13.5)] • Families did not treat drinking water [AOR = 2.85; 95% CI (1.27, 6.42)]
Mosisa D et al. [7]	2021	unmatched case-control	Oromia	• Child’s age (6–11 months) [(AOR 2.46; 95%CI: 1.09–5.57 • Child’s age (12–23 months) (AOR 3.3, 95% CI 1.68–6.46) • Not being vaccinated against rotavirus (AOR 2.45, 95% CI 1.25–4.81)	• Mothers'/caregivers’ history of diarrheal diseases (AOR 7.38, 95% CI 3.12–17.44) • Mothers’/caregivers’ did not wash their hands during critical time (AOR 10.6, 95% CI 3.74–29.81)	• Lack of a hand-washing facility near a latrine (AOR 5.22, 95% CI 3.94–26.49) • Improper domestic solid waste disposal (AOR 2.68, 95% CI 1.39–5.18) • Households who had not utilized latrines properly (AOR 2.34; 95%CI: 1.16, 4.75)
Baye A et al. [16]	2021	Matched case-control	Amhara	• Lack of exclusive breastfeeding (mOR = 2.12; 95% CI: 1.15–3.70)	• Age of mothers/caregivers (>35 years of age) (adjusted matched odds ratio [adjusted mOR] = 2.00; 95% CI: 1.37–5.8) • Marital status (divorced/widowed) [mOR = 1.40; 95% CI: 1.26–3.3] • Mothers/caregivers washed their hands at fewer than three critical times daily (mOR = 4.50; 95% CI: 2.54–9.50)	• Presence of feces within/around latrines (mOR = 1.37; 95% CI: 1.21–3.50) • Lack of handwashing facility near latrine (mOR = 1.50; 95% CI: 1.30–5.30) • Presence of domestic sewage discharge within and/or outside the compound (mOR = 3.29; 95% CI: 1.85–7.50)
Soboksa NE et al. [29]	2020	Matched Case-Control	Oromia	• Number of under-five in the household (being single child) (AOR = 2.76; 95% CI: 1.33–5.71)	• Wealth status (middle (AOR = 5.39; 95% CI: 1.99–14.55) and rich AOR = 3.69; 95%CI: (1.36–10.01) • Mothers/caregivers who did not use soap for hand washing (AOR = 2.89; 95% CI: 1.35–6.15) • Children whose mothers/caregivers do not wash hands before water collection (AOR = 4.28; 95% CI: 1.46–12.56)	• Family collected water from protected well/spring (AOR = 4.01; 95% CI: 1.40–11.44) • Families used the pit as a method of waste disposal (AOR = 4.91; 95% CI: 1.39–13.29)
Girma M et al. [12]	2018	Case-Control	Amhara	• Presence of two or more young siblings (AOR, 4.15; 95% CI: 2.57–6.70) • Rural residence (AOR,2.11 95% CI: 2.21–3.68)	• Lack of hand washing at critical times (AOR, 2.38; 95% CI: 1.42–3.99)	• Unimproved water sources (AOR, 1.88; 95% CI: 1.17–3.03) • Deepening method to take water from a water storage container (AOR, 2.11; 95% CI: 1.28–3.47) • Unsafe child stool disposal (AOR, 1.90; 95% CI: 1.12–3.22)
Brhanemeskel H [63]	2021	Case-control	Addis Ababa	• Bottle feeding (AOR = 3.47, 95% CI: 1.08, 11.16)		
Getachew B et al. [17]	2018	A community-based case-control study	Harari	• Lack of exclusive breastfeeding [AOR: 5.24, 95% CI: (2.46–11.15)] • Consumption of leftover food [AOR: 3.20, 95% CI: (1.26–6.51)]	• Maternal/caregiver’s history of diarrhea [AOR: 4.26, 95% CI: (1.47–12.34)]	• Lacks of home-based drinking water treatment [AOR: 4.27, 95% CI: (2.12–8.60)] • Presence of feces around latrine [AOR: 3.74, 95% CI: (1.88–7.44)]
Alemayehu M et al. [19]	2020	Community based CS	SNNPR	• Unvaccinated for “rotavirus” (AOR: 2.87, 95% CI: 1.86–4.44)	• Mother’s or caretaker’s diarrhea history in the last 2 weeks (AOR: 6.74, 95% CI: 2.51–18.07)	• Non-availability of latrine (AOR: 2.77, 95% CI: 1.66–4.63) • Faeces seen around the pit hole or floor of latrine (AOR: 2.92, 95% CI: 1.38–6.19) • Improper kitchen waste disposal (AOR: 2.31, 95% CI: 1.26–4.24) • Unprotected drinking water source (AOR:1.81, 95% CI: 1.14–2.88)
Hailu B et al. [33]	2021	Community based CS	Amhara	• Playing with soil (AOR = 8.40; 95% CI: 4.58–36.66) • The habit of eating soil (AOR = 6.24; 95% CI: 1.99–19.78)	• Poor handwashing habit after visiting toilet (AOR = 7.70; 95% CI: 2.71–21.79) • Not washing hands before feeding the child (AOR = 19.10; 95% CI: 5.46–66.52)	• Absence of latrine (AOR = 3.87; 95% CI: 1.24–12.08)
Tesfaye TS et al. [34]	2020	Community-based CS	SNNPR		• The number of family members (AOR: 2.7, 95% CI [1.28–5.72]) • Poor hand washing practice during a critical time (AOR: 2.68, 95% CI [1.14–6.32])	• Presence of animals in households (AOR: 2.59, 95% CI [1.19–5.65]) • Absence of latrine (AOR: 2.13, 95% CI [1.01–4.50])
Soboksa NE et al. [50]	2021	Comparative cross-sectional study	Oromia	• In CLTS kebeles: number of under-five children (≥2), (AOR: 3.36; 95%CI: 1.35–8.34)		• Families did not treat drinking water at home compared to those who treated in CLTS kebeles (AOR = 2.35; 95% CI: 1.02–5.98) • In non-CLTS kebeles: family size (≥5), (AOR: 2.93, 95%CI: 1.32–6.49); use of paper (AOR: 2.78, 95%CI: 1.41–8.34), and leaf (AOR:4.58, 95%CI: 2.01–16.47) as anal cleanse material
Getahun W et al. [41]	2021	Community-based CS	Amhara		• Poor handwashing practice at critical times (AOR = 1.85; 95% CI: 1.34–3.56) • No information about diarrhea being prevented by handwashing (AOR = 3.12; 95% CI: 1.64–6.27)	• Water consumption of less than 20 L *per capita* per day ([AOR] = 2.45; 95% CI: 1.36–5.84) • Practicing unsafe child feces disposal (AOR = 2.51; 95% CI: 1.69–4.64) Unimproved sanitation facility (AOR = 3.57; 95 %CI: 1.64–6.51)
Dagnew AB et al. [38]	2019	A community-based CS	Amhara	• Not breastfeeding (AOR = 2.3 (1.023, 5.46)	• Poor hand washing practice (AOR = 6.104 (2.100, 17.738)	• Lack of hand washing facilities in the household (AOR = 3.910 (1.770, 8.634) • Lack of separate feeding materials (AOR = 5.769 (1.591, 9.220)
Getachew A et al. [51]	2018	A community-based CS	Amhara	• Children age (≤1 year) [AOR = 1.82, 95% CI(1.39, 4.63)] • Not breastfeeding [AOR = 3.13, 95% CI(1.62, 6.03)]	• Unable to read and write [AOR: 4.37, 95%CI:1.72–11.08)	
Gebrezgiabher BB et al. [35]	2019	Comparative cross-sectional study	Tigray		• Mothers who had diarrhea in the 2 weeks preceding the survey (AOR = 3.3; 95% CI 1.97, 5.57)	• Non-model households (AOR = 1.9; 95% CI 1.01, 3.56) • Have proper waste disposal method (AOR = 2.6; 95% CI 1.25, 5.58) • Families who did not use latrine (AOR = 2.1; 95% CI 1.128, 3.897)
Wasihun AG et al. [42]	2018	A community based CS	Tigray	• Child age (36–47 months) [AOR = 2.57; 95% CI: 1.45–4.55]	• Mothers not hand washing at critical times [AOR = 15.42; 95% CI: 2.02–117.78]	• Improper solid waste disposal [AOR = 12.81; 95% CI: 2.50–65.62] • Unimproved source of drinking water [AOR = 3.69; 95% CI: 2.03–6.71]
Ayalew AM et al. [52]	2018	Comparative cross-sectional study	Amhara			• In ODF kebeles: water shortage (AOR: 8.75; 95% CI: 1.13–67.83) • In OD kebeles: water shortage (AOR: 18.47; 95% CI: 4.69–72.76)
Adane M et al. [30]	2018	Community based CS	Addis Ababa		• Household size of six or more persons (AOR 2.3; 95% CI 1.4–3.9) • Low monthly household income (AOR 2.4; 95% CI 1.4–4.0)	•
Melese B et al. [24]	2019	Community based CS	Sidama	• Age (12–24 months) [AOR: 12, 95%CI: 1.78, 83.18] • Being under nourished [AOR: 6.41, 95% CI (2.47, 16.77.)]	• Mother with no formal education [AOR: 3.97; 95%CI: 1.60, 8.81] • Not washing hands [AOR, 3.10, 95% CI (1.10, 8.67)] • Use of water only for hand washing [AOR: 6.41; 95%CI (2.51, 16.39)] • Hand washing after visiting latrine [AOR: 2.73, 95% CI (1.05, 6.56)]	• Housing floor material (mud) [AOR: 3.22, 95% CI (1.16, 8.91] • Improper way of refuse disposal [AOR, 3.23, 95% CI (1.37, 7.60)]
Shumetie G et al. [14]	2018	Community based CS	Amhara	• No receipt of Rotavirus vaccine dose 2 [AOR = 3.96, 95%CI; 2.13, 7.33] • Non-exclusive breastfeeding [AOR = 2.69, 95%CI; 1.39, 5.19]	• employed and private business occupational status of mothers [AOR = 2.10, 95%CI; 1.02, 4.31)] • Low household monthly income (less than Ethiopia Birr (ETB) 600) [AOR = 2.10, 95% CI; 1.2, 7.2]	• Unavailability of solid waste disposal system [AOR = 2.62, 95%CI; 1.19, 5.77]
Mekonnen GK et al. [36]	2019	Comparative cross-sectional study	Gambella	• Child’s age (≤5 months), (AOR: 2.46, 95%CI: 1.26–4.81), 6–11 months (AOR: 2.09, 95%CI: 1.15, 3.79)	• Caregiver with no formal education (AOR: 2.59, 95%CI: 1.43, 4.71), primary school (AOR: 1.91, 95%CI: 1.03, 3.55)	• Use of surface water (AOR: 1.92, 95%CI: 1.24, 2.98) • Lack of latrine facility (AOR: 1.45, 95%CI: 1.07, 1.96)
Degebasa MZ et al. [53]	2018	Comparative cross-sectional study	Addis Ababa	• CLTSH implemented: number of undef-five children (≥2), (AOR:2.33, 95%CI: 1.09, 4.96)	• CLTSH implemented: mothers/caregivers had negative attitude toward diarrhea (AOR = 2.07; 95% CI: 1.06–4.04)	• CLTSH implemented: lack of clean water storage (AOR: 2.36, 95%CI: 1.16, 4.80) • Not CLTSH implemented kebeles: Feces in compound (AOR: 1.88, 95%CI: 1.1, 3.22), lack of hand washing facility (AOR: 2.64, 95%CI: 1.47, 4.74)
Mernie G et al. [40]	2022	Comparative cross-sectional study	Amhara		• In CLTSH-implementing areas, use of only water to wash hands (AOR: 3.28; 95% CI:1.13–9.58) and having a mother/caregiver who did not wash their hands at critical times (AOR: 3.02; 95% CI:1.12–8.12) were factors significantly associated with acute diarrhea • From the pooled analysis, not washing hands at critical times (AOR: 2.54; 95% CI:1.59–4.06)	• In non-CLTSH-implementing areas, unimproved water source ([AOR]: 2.81; 95% CI:1.65–4.78), unsafe disposal of child feces (AOR: 2.10; 95% CI:1.13–3.89), improper solid waste disposal (AOR: 1.95; 95% CI:1.12–3.38), and untreated drinking water (AOR: 2.33; 95% CI:1.21–4.49) were factors significantly associated with acute diarrhea • From the pooled analysis, unsafe disposal of child feces (AOR: 2.20; 95% CI:1.34–3.60) and unimproved water source (AOR: 2.56; 95% CI:1.62–4.05)
Wagari S et al. [54]	2022	CS	Oromia	• Child’s age [6–11months, (adjusted prevalence ratio (APR) = 1.54; 95% CI: 1.03–2.29) and 12–23months, (APR = 1.58; 95% CI: 1.01–2.46)		
Feleke Y et al. [15]	2022	CS	Oromia	• Child’s age [7–11months, AOR: 9:15, 95% CI (2.05, 40.71)], and 12–23 months [AOR: 4.98 (1.21, 20.56)] • No of under five children (two and above) [AOR: 2.20; 95%CI: (1.08, 4.49)] • Not exclusive breastfeeding history [AOR: 4.72, 95% CI (1.17, 19.13)]	• Total family size (>5) [AOR: 5.042, 95% CI (2.326, 10.931)] • Washing hands with water only (AOR: 2.95; 95%CI 1.34, 6.48)	
Fufa WK et al. [55]	2019	Comparative cross-sectional study	Oromia	• In urban households: Being male (AOR: 2.3; 95%CI:1.2, 4.7)	• In urban households: Poor knowledge on home management of diarrhea (AOR = 2.7(CI [1.3, 6.5]), difficulty in preparing oral rehydration salt (AOR = 4.0CI [1.4, 11.0) • In rural households: Poor knowledge on home management of diarrhea AOR = 13.4(CI [5.3, 34.0]), difficulty in preparing oral rehydration salt (AOR: 2.4; 95%CI:1.3, 5.3), easy to get Zinc (AOR: 2.4; 9%CI: 1.2, 5.0)	
Feleke DG et al. [56]	2022	Community based CS	Amhara	• Initiation of supplementary food before 6 months (AOR = 6.49 (2.01–20.96))		• Households with unclean latrine (AOR = 11.48 (5.64–23.35)) • Lack of hand washing facility (AOR: 7.07; 95%CI 3.84–13.03) Lack of solid waste disposal pit (AOR: 1.78, 95%CI: 0.95–3.36)
Megersa S et al. [57]	2019	Comparative cross-sectional study	Oromia		• In non ODF Sub-districts: age (12–23 months) (AOR: 3.71, 95%CI: 1.50, 9.19)	• In non ODF Sub-districts: Presence of faces in the compound (AOR: 2.10; 95%CI: 1.05, 4.17)
Beyene SG et al. [58]	2018	Community based CS	Oromia			• Living in the home with single room [(AOR = 6.01, 95% CI(1.01, 36.01)] • Living with animal in the same house [AOR: 8.31, 95% CI(2.46, 28.06)]
Kasee LF et al. [59]	2018	Community-based CS	Oromia	• *No regression analysis*		
Gashaw TA et al. [23]	2019	Community- based CS	SNNPR	• Being underweight (AOR: 3.14; 95%CI: 1.93, 5.11) • Not received full vaccination (AOR: 2.06, 95%CI: 1.021, 4.16)	Mother education level (no education [(AOR: 3.89; 95%CI: 1.62–9.36)]	
Bitew BD et al. [31]	2022	Community-based CS	Amhara	• Child’s age (1–12months) [AOR: 9.22, 95%CI: (2.93–29.04)] and age group of 13–24 months [AOR: 4.44, 95%CI: (1.87–10.56)]	• Low monthly income (AOR: 3.68, 95% CI: (1.81–7.51)] Poor hand washing practice [AOR: 8.37, 95% CI: (3.12–22.52)	
Getachew F et al. [60]	2022	Community-based CS	Addis Ababa	•	• Low household income (<1000 ETB)[AOR: 7.21, 95% CI (1.49, 34.92)] • Maternal history of diarrhea [AOR: 8.03, 95% CI (1.32, 48.67)]	• Water storage duration (1 week and above) [AOR: 5.10, 95% CI (1.47, 17.62)] • Lack of hand washing facility [AOR: 5.70, 95% CI (1.01, 32.247)] Presence of uncollected garbage in the compound [AOR: 3.42, 95% CI (1.38, 8.49)]
Zedie FB et al. [28]	2018	Comparative cross-sectional study	Sidama	• Child’s age (12–23 months) [AOR: 3.74, 95%CI (1.23, 10.53)]	• Mothers with self or private employed [AOR: 5.08, 95%CI (1.88, 13.66)] • Mothers used soap sometimes when washing hands [AOR: 2.37, 95%CI (1.06, 5.27)], or did not use at all [AOR: 33.33, 95%CI (13.58, 76.98)]	• Non- HDA (health development army) [AOR: 1.88, 95%CI (1.05, 3.37)] • Distance of latrine from the house [6 to 10, (AOR: 2.63, 95%CI: 1.16, 5.97), and above 10 m (AOR: 3.22, 95%CI (1.26, 8.24)] • Households that had no separate kitchen [AOR: 3.42, 95%CI (1.77, 6.62)] • Not treat water at home [AOR: 12.88 95%CI (1.42, 16.57)]
Chomissa AR et al. [20]	2018	A community based comparative cross-sectional	SNNPR	• Unvaccinated for Rota vaccine (AOR = 2.45; 95%CI: 1.48–4.04)	• Mothers who had history of diarrhea (AOR = 2.32; 95%CI: 1.28–4.17)	• Non-model households (AOR = 2.54; 95%CI: 1.55–4.17) • Households who did not have latrine (AOR: 3.07; 95%CI: 1.62–5.86) • Unsafe child stool disposal (AOR = 2.19; 95%CI: 1.32–3.64)
Arba A et al. [89]	2020	A community based cross-sectional	SNNPR	• -	• -	• -
Amamo DD et al. [62]	2019	A community based cross-sectional	Oromia	• Child’s age [6–11 months [AOR = 2.72; 95% CI(1.18, 6.27)], 12–23 months [AOR: 2.28; 95%CI (1.13, 4.62)], 24–35 months (AOR: 2.13; 95%CI: (1.21, 3.75)] • Number of under five children (two) [AOR = 1.527; 95% CI: (1.04, 2.24)] • Not exclusive breastfeed [AOR = 2.45; 95% CI: (1.61, 3.73)] • Early initiation of supplementary feeding (<6 months) [AOR = 2.16; 95% CI(1.22,.3.83)] • Not received pneumococcal vaccination [AOR = 6.72; 95% CI(1.20,.37.65)] • Not received Vitamin A supplementation [AOR = 1.66: 95% CI(1.04,.2.68)]	• Maternal educational status (primary education) [AOR = 3.75, 95% CI: (1.07, 13.22)]	• Improper waste disposal method [AOR = 1.92; 95% CI: (1.26, 2.94)]
Zegeye Z [90]	2021	A community based cross-sectional	Amhara		• Family size (≥5 members (AOR = 2.48, 95%CI: 1.58, 3.89) • Use of water for hand washing (AOR = 3.45, 95%CI: 2.20, 5.41)	Feacal mater seen around the pit-whole (AOR = 1.86, 95%CI: 1.19, 2.91)
Mengistu KD [61]	2021	Health center based cross-sectional study	Amhara	• Child feeding (children mostly taking adult food [AOR = 6.42 (1.09, 37.53)] • Duration of breastfeeding (<6 months) [AOR = 7.64 (1.51, 38.63) • Start supplementary feeding before less than 6 months [AOR = 9.764 (95% CI, 1.79, 53.071)] • Not taking measles vaccination [AOR = 4.78 (1.813, 12.647)]	• Low monthly income (below 3874 ETB) [AOR = 19.45 (1.439, 263.059)]	
Angasu K et al. [6]	2022	Institution based cross-sectional study	SNNPR, Addis Ababa, and Oromia	• Child’s age (12–23 months) [AOR 4.37; 95%CI = 2.32–8.24] • Child feed by her/his own (AOR 2.4; 95%CI = 1.57–3.74)	• Mothers or guardian with no formal education (AOR 3.55; 95%CI = 1.97–6.4)	• Washing child utensils by cool-water and soap (AOR 2.8; 95%CI = 2.12–3.57) Unprotected water (AOR 3.6; 95%CI = 2.13–6.13)
Mitiku HD [13]	2021	A community based cross-sectional study	Amhara	• Rural residency (AOR: 2.99; 95%CI: 1.30, 6.90) • Child not lived with biological mother (AOR:32.44, 95%CI: 14.07, 74.80) • Child live with currently pregnant of mothers (AOR: 5.66; 95%CI: 2.67, 11.99) • Not receiving measles vaccination (AOR: 3.91; 95%CI: 2.26, 6.77)	• Not washing hands after visiting toilet (AOR = 7.91, 95% CI, 2.77, 22.59)	• Unprotected sources of drinking water (AOR: 14.01; 95%CI: 7.50, 26.15) • Sub-standard toilet facility (AOR: 2.74; 95%CI: 1.27, 5.89)
Kassie G [91]	2020	A community-based cross-sectional study	Amhara			
Alemayehu B et al. [21]	2020	Community-based CS	Southwest Ethiopia	• Childs’s age (<6 months) (AOR 2.5; 95% CI 1.23–4.49) • Unvaccinated children for rotavirus prevention (AOR: 5.22, 95%Ci: 3.33–8.20)		• Sharing of the residence with domestic animals (AOR: 2.87, 1.75–4.67) • Households obtaining water from unimproved sources (AOR: 2.53; 95%CI: 1.60–4.40)
Natnael T et al. [9]	2021	Community based CS	Amhara	• Child’s age (12–23 months) (AOR: 4.68, 95% CI: 1.45–1.50) • Presence of two or more under-five children in the house (AOR = 2.84, 95% CI: 1.19–6.81)		• Unimproved water sources (AOR = 2.97, 95% CI: 1.28–6.87) • Presence of feces around the pit hole/slab/floor of the latrine (AOR = 3.34, 95% CI: 1.34–8.31)
Bekele D et al. [22]	2021	A community-based comparative cross-sectional study	Oromia	• Household health extension program implementation: Not vaccinated against Rotavirus (AOR: 49.8; 95% CI: 4.2–94.8); child not supplemented with vitamin A (AOR: 3.2; 95%CI:1.4–7.2) • Non-model families: child not vaccinated against Rotavirus (AOR: 10.9; 95%CI: 2.9–41.1)	• Non-model families: Family size >5 (AOR [95% CI] = 5.2 [1.7–17.6])	• Household health extension program implementation: model household (AOR: 2.4; 95%CI: 1.15, 5.00) • Unimproved water sources (AOR: 5.5; 95%CI: 2.2,-97.7) Non-model families: Unimproved water sources (AOR [95% CI] = 7.2 [1.6–13.2]), not using latrine (AOR [95% CI] = 6 [1.8–20.6])
Fenta A et al. [43]	2020	A community-based CS	Beni Shangul Gumuz	• Complementary feeding before 6 months (AOR = 6.49, 95%CI: 2.01–20.96)	• Poor handwashing practice at a critical time (AOR = 5.92, 95%CI: 2.58–13.70)	• Poor latrine hygiene (AOR = 11.48, 95%CI: 5.64–23.35) • No handwashing facilities near latrines (AOR = 7.07, 95%CI:3.84–13.03) • Water storage (AOR = 8.6, 95%CI: 1.51–48.84)
Alemayehu K et al. [8]	2021	A community based CS	Oromia	• Child’s age: 6–11 months, (AOR: 1.55; 95%CI: 1.68, 3.52), and 12–23 months (AOR: 1.48; 95%CI: 1.84, 2.63) • Children who were not vaccinated against measles (AOR: 4.73, 95% CI: 2.43, 9.20) • Having two or more siblings (AOR: 3.11, 95% CI (1.81, 5.35)	• Poor knowledge of mothers/caretakers on diarrhea prevention methods (AOR: 2.05, 95% CI (1.14, 3.69) Poor wealth index (AOR: 2.41, 95% CI (1.29, 4.51)	• Inappropriate liquid waste disposal (AOR: 3.73; 95%CI: 1.94, 7.42) • Unsafe child feces disposal (AOR: 3.75; 95% CI (1.91, 7.39)
Shine S et al. [18]	2020	A community-based CS	Amhara	• Child’s age (7–11 months) (AOR: 4.2, 95% CI: 1.2–15.3) • Being the second-born child (AOR: 3.9, 95%CI: 1.8–8.5) • Not vaccinated against rotavirus (AOR: 10.3, 95%CI: 3.2–91.3)	• Feeding children by hand (AOR: 2.5, 95%CI: 1.1–6.1)	
Solomon ET et al. [44]	2020	A community-based CS	Dire Dawa		• Maternal diarrhea (AOR = 2.22, 95% CI 1.10–4.47) • Not handwashing after contact with child feces (AOR = 6.27, 95% CI 2.01–19.55)	• Use of a dipper to draw water from containers (AOR = 2.88, 95% CI 1.41–5.89) • Lack of refuse disposal facility (AOR = 2.47, 95% CI 1.09–5.60)
Tafere Y et al. [26]	2020	A community-based comparative cross-sectional study	Amhara	• Age of the child (>12 months) (AOR: 2, 95% CI (1.4, 2.7)	• Not attending formal education (AOR: 2.1, 95% CI (1.2, 2.7) • Family size ≥5 (AOR: 1.3, 95% CI (1.11, 1.9) • Low monthly income (AOR: 2.1, 95% CI (1.3, 2.) • Mothers had a diarrheal diseases in the last 2 weeks (AOR: 1.2; 95%CI: 1.1, 24) • Not handwashing after touching infants’ faeces (AOR:1.6; 95%CI: 1.12, 2.03)	• Status of kebeles NODF [AOR: 2.4; 95%CI: (1.17, 3.23)] • Number of rooms in the house (1–2 rooms) [AOR: 1.3; 95%CI (1.1, 1.7)] • Type of house roof constructed (grass) [AOR: 2.3; 95%CI: (1.53, 3.4)] • Latrine utilization pattern [Sometimes, AOR: 1.50; 95%CI(1.1, 2.4)] • Lack of functional handwashing facility near the latrine (AOR: 11; 95%CI:8.1, 29.6) • Unsafe child stool disposal (AOR: 1.4; 95%CI: 1.1, 3.7) • Old latrine (>4 years) (AOR: 1.8; 95%CI: 1.2, 3.1) • Faeces seen around the compound 27.6 (18.9, 37)-
Mulu E et al. [32]	2022	Community-based cross-sectional study	SNNPR	• Bottle feeding [AOR: 8.27, 95% (1.09, 62.97)]	• Mothers/care takers who feed adult food to the children [AOR: 6.98, 95% (1.07, 45, 43)]	• Unimproved toilet facility feces seen outside the pit hole of latrines [AOR: 2.94, 95% (1.35, 6.43)] • Unimproved water source [AOR: 4.47, 95% CI (1.96, 10.21)] • Distance to water source (>1 h) [AOR: 2.25, 95% (1.14, 4.45)]

### Child Related Factors

The most consistent factors associated with diarrhea, as shown in Table 3 were the child’s age, feeding status, nutritional status, and vaccination status for rotavirus.

Several studies consistently reported the highest prevalence of diarrhea among children occurred between one and 12 months of age [7, 8, 11, 15, 18, 31, 36, 54, 62]. Studies also found that bottled-fed children [11, 32, 63], not breastfeeding [38, 51], lack of exclusive breastfeeding [14–17], and starting complementary feeding before 6 months [61, 62] were the commonly identified associated factors with childhood diarrheal disease. Moreover, not being vaccinated against rotavirus [7, 14, 18–22], playing with soil and a habit of eating soil [33], being underweight [23, 24], and not receiving Vitamin A supplementation [22, 62] were factors associated with higher risk of diarrheal disease. Three studies found an increased risk of diarrheal disease among children living in households with two and above under-fives [50, 53, 62] (Table 3).

Supplementary Material S4A–F presents the pooled odds ratios of the association between child age, the child’s sex, and childhood diarrhea in Ethiopia. As depicted in Supplementary Material S6A, there is no statistically significant difference in the odds of experiencing childhood diarrheal disease between male and female children under the age of five (pooled OR = 0.61; 95% CI: 0.35–1.07, I^2^ = 88.0%, *p* < 0.001).

The likelihood of childhood diarrhea was 1.79 times higher among children aged 0–5 months (pooled OR = 1.79, 95% CI: 1.05–3.05, I^2^ = 60.8%, *p* = 0.006), 2.40 times higher among children aged 6–11 months (pooled OR = 2.40, 95% CI: 1.77–3.25, I^2^ = 38.3%, *p* = 0.071), and 1.65 times higher among children aged 12–23 months (pooled OR = 1.65, 95% CI: 1.07–2.54, I^2^ = 73.1%, *p* < 0.001) compared with children aged 48–59 months (Supplementary Material S6B–D).

However, there is no statistically significant association observed between diarrhea occurrence among children aged 24–35 months (pooled OR = 1.16, 95% CI: 0.89–1.51, I^2^ = 27.5%, *p* = 0.168) and 36–47 months (pooled OR = 1.15, 95% CI: 0.73–1.80, I^2^ = 62.1%, *p* = 0.015) when compared with those aged 48–59 months (Supplementary Material S6E, F).

### Parental and Household Factors

Across the studies, the lack of maternal education [6, 11, 23, 24, 26, 62], poor maternal knowledge about diarrheal disease and its prevention measures [8, 27], and mothers poor handwashing practices (i.e., not washing their hands at the critical times, such as after visiting toilet, after touching children’s feces) [7, 12, 13, 26, 31, 33, 34, 38–44] were identified associated factors with acute diarrheal disease (Table 3).

This meta-analysis’s findings showed that there was association between mothers not washing their hands after using the toilet with children having diarrhea (pooled OR = 3.05; 95% CI:2.08–4.54, I^2^ = 75.3%, *p* < 0.001, n = 15) (Supplementary Material S7A). Four studies [6, 15, 30, 54] used different reference categories, and we pooled the confounder adjusted odds ratios separately. Similarly, the odds of childhood diarrhea was lower by 51% among children whose mothers washed their hands at the critical time (pooled OR = 0.49, 95% CI: 0.31–0.70, I^2^ = 48.6%, *p* = 0.120, n = 4).

Mothers’ history of diarrheal episodes in the previous 2 weeks before the study was also identified as a risk factor for childhood diarrheal disease [7, 11, 17, 19, 20, 26, 27, 35, 39, 60, 64]. The overall result of the meta-analysis revealed that children whose mothers had a history of diarrhea in the previous 2 weeks were three times more likely to develop diarrhea than children without a history of maternal diarrhea in the previous 2 weeks (pooled OR = 3.19, 95%CI: 1.94–5.25, n = 13). We used a random effect meta-analysis model to estimate pooled OR because the included studies had high heterogeneity (I^2^ = 79.4%, *p*-value = 0.006) (Supplementary Material S7B). Since two studies [28, 54] used different reference categories, we pooled the odds ratios separately. Likewise, the odds of diarrhea was reduced in children whose mothers had not recently experienced any diarrheal episode (pooled OR = 0.49, 95% CI: 0.35–0.68, I^2^ = 0.0%, *p* = 0.901, n = 2).

### Water, Sanitation and Hygiene (WASH) Factors

As shown in Table 3, the most consistent WASH related factors associated with diarrheal disease in Ethiopia were i) the lack of toilet facility or use of unimproved toilet facility [7, 19, 20, 32–36], ii) the poor latrine hygienic condition [9, 16, 17, 19, 26, 43, 56], iii) unsafe child stool disposal [11, 12,19, 20, 26, 27, 39–41], iv) poor domestic solid waste disposal [7, 8,14, 19, 40, 42, 56, 62], v) not treating drinking water [11, 27, 28, 39, 50], vi) collecting water from unimproved drinking sources [6, 9, 12, 13, 19, 21, 32, 40, 42], and vii) lacked handwashing facilities near the toilet facilities [12, 38, 56, 60].

The pooled result of this meta-analysis indicated that children from households that lacked handwashing facilities near the toilet were 4.16 times more likely to have diarrheal morbidity as compared to their counterparts (pooled OR = 4.16, 95%CI: 2.49–6.95, I^2^ = 71.1%, *p* = 0.001, n = 9) (Supplementary Material S7C). Children living in households without latrine facilities were found to be 1.56 times more likely to develop diarrhea than children living in households with latrine facilities (pooled OR = 1.56, 95%CI: 1.05–2.33, I^2^ = 71.1%, *p* = 0.001, n = 9) (Supplementary Material S7D).

The meta-analysis results of ten studies that reported confounder adjusted association between household water treatment and childhood diarrhea. The overall result showed that, children from households that did not use treated drinking water at home were two times higher odds of having diarrheal disease compared to children living in households that used home treated drinking water (pooled OR = 2.28, 95% CI: 1.50–3.46, I^2^ = 64.5%, *p* = 0.003, n = 10) (Supplementary Material S7E).

## Discussion

Childhood diarrheal disease can cause significant morbidity and remain a significant public health issue across the world. This systematic review and meta-analysis aimed to estimate the pooled prevalence of diarrhea disease and summarize the potential associated factors among under-fives in Ethiopia. The pooled prevalence showed that one in every five children under the age of 5 years in Ethiopia experienced diarrheal disease. Overall, early childhood, poor child nutrition status, use of unimproved sanitation, lack of hand washing facilities, maternal diarrhea illness in previous 2 week, inadequate maternal hygiene knowledge and behavior, and improper domestic solid waste disposal were important factors associated with diarrheal disease.

The meta-analysis findings from the included 44 studies indicated that 20.8% of children under five in Ethiopia experienced diarrheal diseases. The results are comparable with the 2016 Ugandan Demographic and Health Survey (DHS) that reported the prevalence of diarrhea among children <5 years in Uganda to be 20% [65]. Our finding was in line with the prior pooled prevalence estimates of diarrhea 22% [37]. Although the results showed a relatively lower prevalence, this figure seemed to imply that the prevalence of diarrheal disease has not dropped significantly. As such, it would be reasonable to argue that diarrheal disease was still a significant public health concern of high magnitude in Ethiopia. On the other hand, our finding showed that the diarrheal disease prevalence was higher than the overall prevalence of diarrheal disease among under-fives in sub-Saharan Africa 15.3% [66] and the East Africa pooled estimate 14.28% [67]. The observed higher prevalence of diarrheal disease in this review compared to the East Africa regional estimate could be due to the differences in methodological approaches. The DHS sampling, design, and setting were very different from the type of analysis that was used in the current study. The results of DHS reports are based on primary data collection, whereas we reviewed and used prevalence measurements from previously conducted primary studies from different areas, populations, and seasons.

The top three regions with the highest prevalence of diarrhea were observed in the Gambella region, 33.0%, SNNPR region 24.21%, and Dire Dawa city administration 23.0%, according to the subgroup analysis of this study. The Sidama region had the lowest proportion of diarrheal disease, at 14.24%. Regional estimates could differ due to differences in basic sanitation, household behavioral characteristics, and access to healthcare facilities. The high prevalence of diarrheal disease in the Gambella region could also be due to the inclusion of a single comparative cross-sectional study in sub-group meta-analysis, which was limited to a single area and did not represent the entire region. Corresponding to the Ethiopia DHS 2016 report, the Gambela region had the highest diarrheal morbidity in Ethiopia (14.5%), followed by the SNNP region (13.9%) [4].

The findings of our review suggest that there is no significant difference in the prevalence of childhood diarrheal disease between male and female children under the age of five. This observation aligns with previous studies that have also failed to identify gender as a significant risk factor for diarrheal illness [68]. Our study adds to the body of evidence indicating that diarrheal disease does not exhibit a gender-specific pattern among young Ethiopian children. Our review also highlights a notably high prevalence of diarrheal disease among children aged 12–23 months. Several factors may contribute to the heightened susceptibility in this age group, including increased mobility and exploratory behavior, which lead to greater exposure to contaminated environments [69].

The current study found lack of exclusive breastfeeding to be associated with a higher likelihood of diarrheal disease in children [14–17]. Evidence showed that breastfeeding is an ideal food for infants and young children and it prevents nearly half of all diarrheal episodes and 72% of diarrheal-related hospitalizations [70, 71]. Breast milk is safe, clean, and contains antibodies which help to protect children against many common childhood illnesses [72]. A meta-analyses of eighteen studies showed that not breastfeeding resulted in an excess risk of diarrheal mortality in comparison to exclusive breastfeeding among infants 0–5 months of age and to any breastfeeding among children aged 6–23 months [70]. This study identified significant association between being underweight [26, 27] and wasting [14] and childhood diarrhea. The relationship between child undernutrition and diarrheal illnesses was clearly shown [73] as diarrheal disease and malnutrition being bidirectionally related, meaning that: diarrhea causes malnutrition, and malnutrition worsens the course of diarrheal disease [74].

Across the studies, lack of maternal education was consistently associated with higher odds of childhood diarrheal [6, 11, 23, 24, 26, 62], which is consistent with research conducted in Nigeria [75]. The educational attainment of mothers influences hygienic practices, child feeding, weaning, and sanitation practices, all of which are crucial factors against the onset of childhood diarrheal disease. Primary studies identified mothers’ poor handwashing practices [7, 12, 13, 26, 31, 33, 34, 38–44] and mothers’ history of diarrheal episodes in the previous 2 weeks [7, 11, 17, 19, 20, 26, 27, 35, 39, 60, 64] as potential factor associated with childhood diarrhea. The result of this meta-analysis revealed that, the odds of childhood diarrhea was three times higher among children whose mothers did not practice hand washing at critical times. Likewise, the odds of childhood diarrhea among children whose mother had a history of diarrhea in the previous 2 weeks was three times higher than their counterparts. The importance of proper handwashing at critical times with appropriate handwashing agents has long been established in the reduction of diarrheal disease with handwashing with soap reducing the burden significantly [76–78]. For instance, a recent systematic review, handwashing promotion in communities prevents one-quarter of diarrhea episodes in Low and Middle-Income Countries (LMICs) [79]. The pooled result of this meta-analysis indicated that children from households that lacked handwashing facilities near the toilet were four times more likely to have diarrheal morbidity as compared to their counterparts. This finding supports previous studies which have identified lack of a handwashing station as predictors of diarrheal disease [12, 38, 56, 60]. This finding was not surprising as several studies explore strong link between handwashing and childhood diarrhea [80]. A systematic review of the literature by Shah et al. also reported handwashing are effective strategy for preventing all causes of diarrheal disease [81].

Children living in households that used untreated drinking water and those collecting water from unimproved drinking water sources were at a higher risk of diarrheal disease. Several studies provided evidence that improving access to safe drinking water reduces the risk of diarrheal disease in children [25, 82]. In our meta-analysis, the odds of developing childhood diarrhea in households that did not use treated drinking water at home were two fold higher compared to children used home treated drinking water, and that improved water quality was generally is an effective strategy in preventing diarrheal disease [83]. Although evidence exists that improved sanitation is one of the key factors in the reduction of diarrheal diseases [52, 84], however, to establish the effect of improved latrines on diarrheal disease prevention in the absence of universal or at least adequate latrine coverage in a given community is controversial and difficult as indicated in the recent cluster-randomized controlled trial on sanitation interventions reporting that improved latrines had no protective effect against childhood diarrheal prevalence [85, 86]. However, other systematic review and meta-analysis have shown that both improved neighborhood sanitation conditions and household sanitation are associated with reduced diarrheal illness [87].

### Limitations

First, in the majority of the included studies diarrheal disease was based on self-reported screening and was not further clinically confirmed. Second, our study could lack representativeness at a country level as we did not find a study from some regions of Ethiopia. Third, there was high heterogeneity between included studies, as indicated by the I^2^ statistic. Fourth, we were unable to pool all the adjusted odds ratio as a number of the included studies showed significant heterogeneity and used different reference category. As a result, this work only summarizes the findings as described in the study protocol. Fifth, because the studies in this review were all observational in design, it is possible that other confounding variables could influence the identified associated factors. Sixth, the majority of included articles were observational studies (i.e., cross-sectional studies), so causality cannot be inferred. Last but not least, because our search was restricted to publicly accessible databases, we were unable to include databases like EMBASE.

### Conclusion

Our study indicated that approximately one in five under-five children in Ethiopia experienced diarrheal disease during the 2 weeks, and there were regional variations in diarrheal prevalence among under-five children. The key contributing factors to childhood diarrheal were related to child, parental, and WASH factors (such as poor handwashing practices, use of untreated drinking, household that lacked handwashing facility near the toilet facility, and lack of toilet facility). To address diarrheal disease and achieve the Sustainable Development Goals and the 2030 agenda [88], Ethiopia must strive to strengthen current strategies and consider the identified factors. To reduce diarrheal disease among children under the age of five, priority should be given to strengthening interventions that focus on improving household WASH facilities and raising awareness about the importance of handwashing, proper sanitation, and hygiene practices.
