# Supplementary material for: Burden of Childhood Diarrhea and Its Associated Factors in Ethiopia: A Review of Observational Studies
Source: Int J Public Health. 2024 Jun 5;69:1606399. doi: 10.3389/ijph.2024.1606399 (PMC11188320; doi:10.3389/ijph.2024.1606399)
Supplement: Supplementary file 1 [file DataSheet4.docx]

**Supplementary file 4 (a):** Pooled prevalence of diarrhea among under-five children in age group of 0-5 months in Ethiopia.

**Supplementary file 4 (b):** Pooled prevalence of diarrhea among under-five children in age group of 6-11 months in Ethiopia.

**Supplementary file 4 (c):** Pooled prevalence of diarrhea among under-five children in age group of 12-23 months in Ethiopia.

**Supplementary file 4 (d):** Pooled prevalence of diarrhea among under-five children in age group of 24-59 months in Ethiopia.
