# Supplementary material for: Burden of Childhood Diarrhea and Its Associated Factors in Ethiopia: A Review of Observational Studies
Source: Int J Public Health. 2024 Jun 5;69:1606399. doi: 10.3389/ijph.2024.1606399 (PMC11188320; doi:10.3389/ijph.2024.1606399)

**Supplementary file 7 (a):** The pooled odds ratio of the association between maternal hand washing practices and childhood diarrhea in Ethiopia.


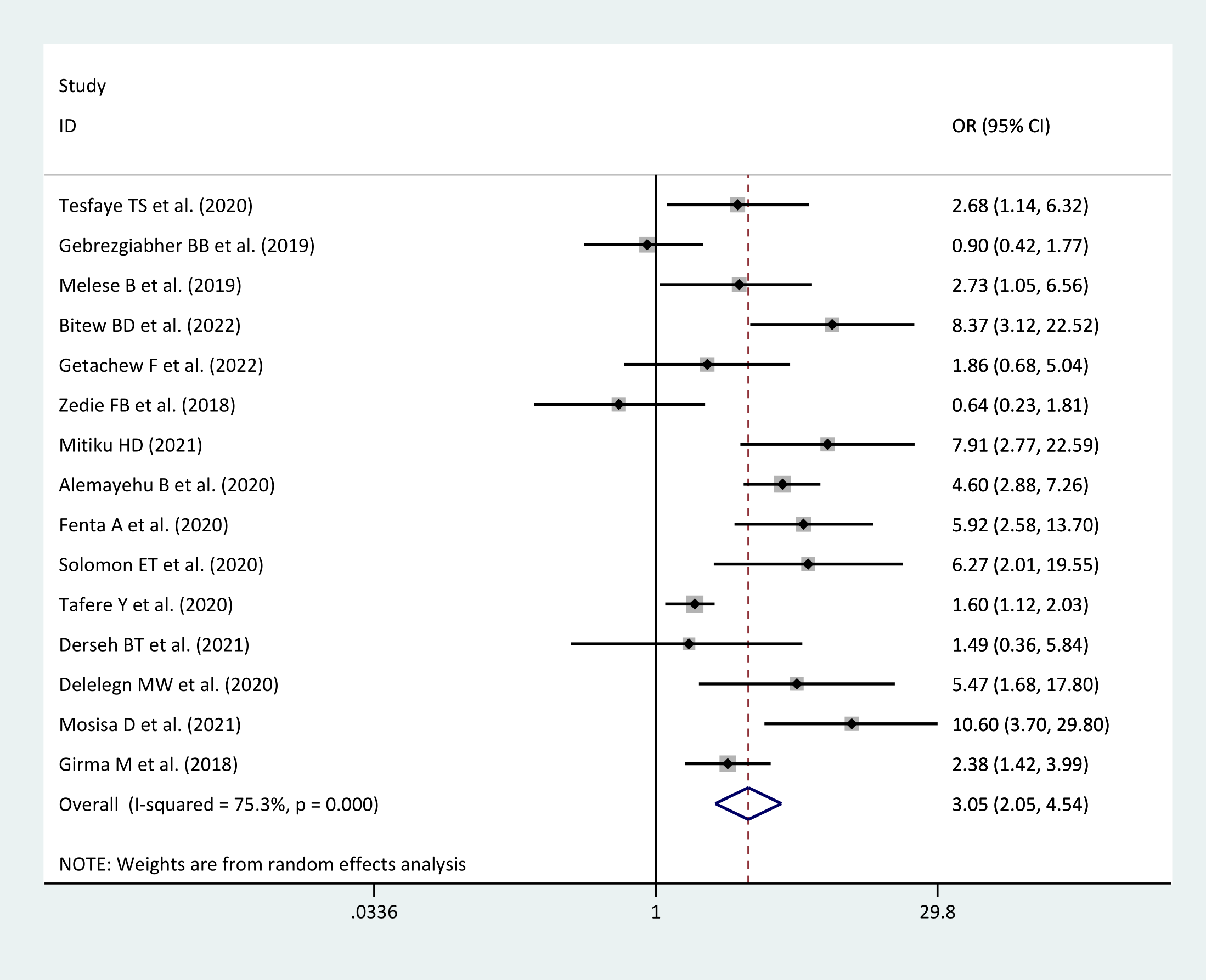


**Supplementary file 7 (b):** The pooled odds ratio of the association between mothers who had a history of diarrhea and childhood diarrhea in Ethiopia.


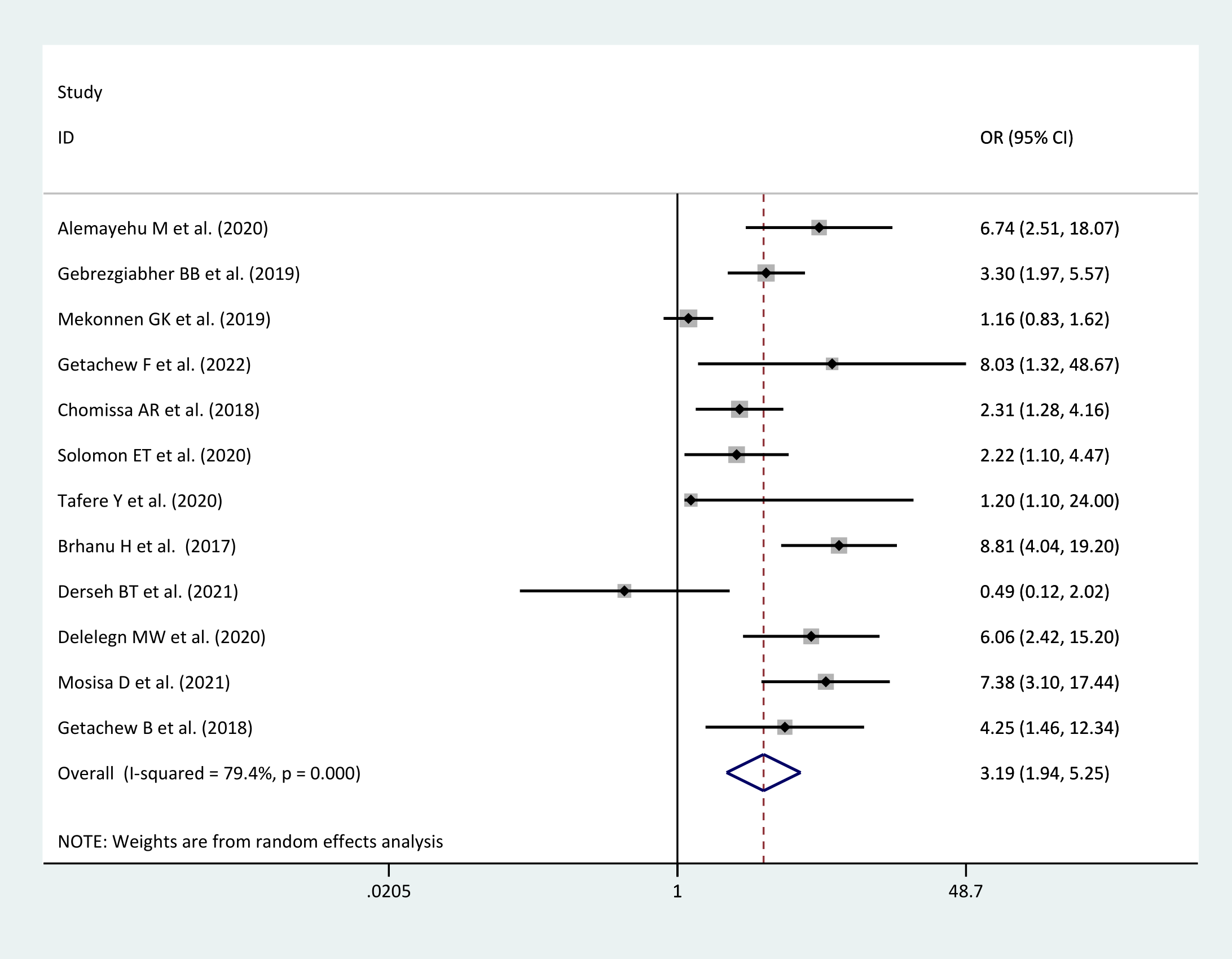


**Supplementary file 7 (c):** The pooled odds ratio of the association between households that lacked handwashing facilities near the toilet facilities and childhood diarrhea in Ethiopia.


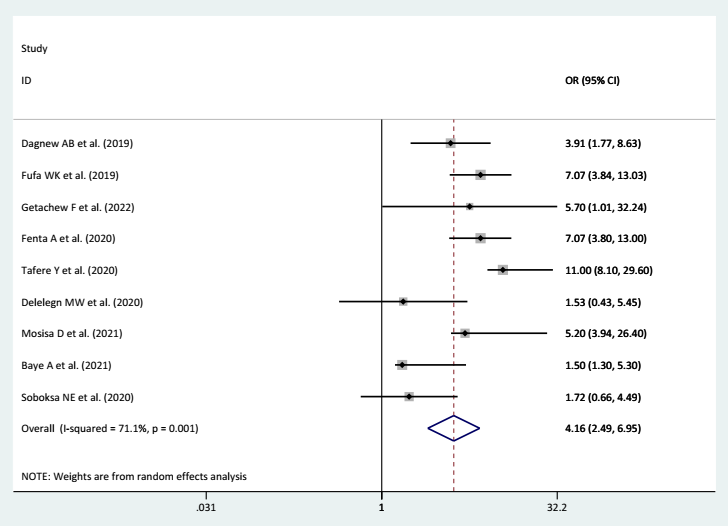


**Supplementary file 7 (d):** The pooled odds ratio of the association between latrine availability and childhood diarrhea in Ethiopia.


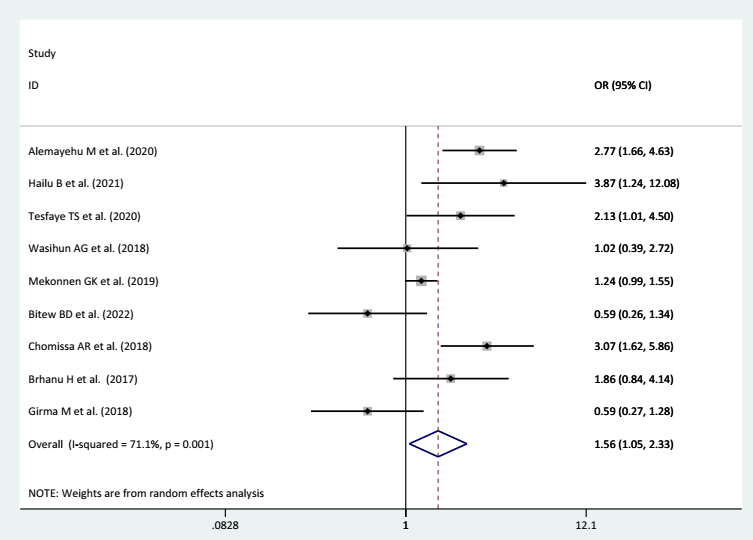


**Supplementary file 7 (d):** The pooled odds ratio of the association between household water treatment and childhood diarrhea in Ethiopia


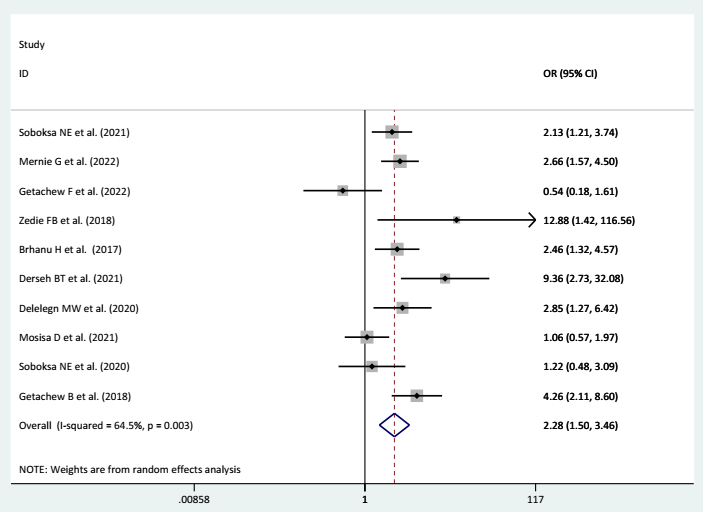

Supplement: Supplementary file 2 [file DataSheet7.docx]
