# Supplementary material for: Burden of Childhood Diarrhea and Its Associated Factors in Ethiopia: A Review of Observational Studies
Source: Int J Public Health. 2024 Jun 5;69:1606399. doi: 10.3389/ijph.2024.1606399 (PMC11188320; doi:10.3389/ijph.2024.1606399)
Supplement: Supplementary file 3 [file DataSheet6.docx]

**Supplementary file 6 (a):** The pooled odds ratio of the association between sex of the child and childhood diarrhea in Ethiopia.

**Supplementary file 6 (b):** The pooled odds ratio of the association between child age (0-5 months) and childhood diarrhea in Ethiopia.

**Supplementary file 6 (c):** The pooled odds ratio of the association between child age (6-11 months) and childhood diarrhea in Ethiopia.

**Supplementary file 6 (d):** The pooled odds ratio of the association between child age (12-23 months) and childhood diarrhea in Ethiopia.

**Supplementary file 6 (e):** The pooled odds ratio of the association between child age (24-35 months) and childhood diarrhea in Ethiopia.

**Supplementary file 6 (f):** The pooled odds ratio of the association between child age (36-47 months) and childhood diarrhea in Ethiopia.
