# Supplementary material for: Burden of Childhood Diarrhea and Its Associated Factors in Ethiopia: A Review of Observational Studies
Source: Int J Public Health. 2024 Jun 5;69:1606399. doi: 10.3389/ijph.2024.1606399 (PMC11188320; doi:10.3389/ijph.2024.1606399)
Supplement: Supplementary file 4 [file DataSheet3.docx]

**Supplementary file 3**

**Table 1: Critical appraisal checklist for observational studies (Joanna Briggs Institute) in the systematic review according to the 10 items (*for cross-sectional studies*)**

|  | **Author name** | **1** | **2** | **3** | **4** | **5** | **6** | **7** | **8** | **Final score** | **Overall Appraisal** |
| --- | --- | --- | --- | --- | --- | --- | --- | --- | --- | --- | --- |
| 1 | Alemayehu M et al. (22) | Y | Y | N | Y | N | Y | N | Y | 5/8 | Included |
| 2 | Hailu B et al. (36) | Y | Y | N | Y | N | Y | N | Y | 5/8 | Included |
| 3 | Tesfaye TS et al. (37) | Y | Y | N | Y | N | Y | N | Y | 5/8 | Included |
| 4 | Soboksa NE et al. (55) | Y | Y | N | Y | N | Y | N | Y | 5/8 | Included |
| 5 | Getahun W et al. (43) | Y | Y | N | Y | N | Y | N | Y | 5/8 | Included |
| 6 | Dagnew AB et al. (40) | Y | Y | N | Y | N | Y | N | Y | 5/8 | Included |
| 7 | Getachew A et al. (54) | Y | Y | N | Y | N | Y | N | Y | 5/8 | Included |
| 8 | Gebrezgiabher BB et al. (38) | Y | Y | N | Y | N | Y | N | Y | 5/8 | Included |
| 9 | Wasihun AG et al. (44) | Y | Y | N | Y | N | Y | N | Y | 5/8 | Included |
| 10 | Ayalew AM et al. (91) | Y | Y | N | Y | N | N | N | Y | 4/8 | Included |
| 11 | Adane M et al. (33) | Y | Y | N | Y | N | Y | N | Y | 5/8 | Included |
| 12 | Melese B et al. (27) | Y | Y | N | Y | N | Y | N | Y | 5/8 | Included |
| 14 | Shumetie G et al. (17) | Y | Y | N | Y | N | Y | N | Y | 5/8 | Included |
| 15 | Mekonnen GK et al. (39) | Y | Y | N | Y | N | Y | N | Y | 5/8 | Included |
| 16 | Degebasa MZ et al. (56) | Y | Y | N | Y | N | Y | N | Y | 5/8 | Included |
| 17 | Mernie G et al. (42) | Y | Y | N | Y | N | Y | N | Y | 5/8 | Included |
| 18 | Wagari S et al. (51) | Y | Y | N | Y | N | Y | N | Y | 5/8 | Included |
| 19 | Feleke Y et al. (18) | Y | Y | N | Y | N | Y | N | Y | 5/8 | Included |
| 20 | Fufa WK et al. (52) | Y | Y | N | Y | N | Y | N | Y | 5/8 | Included |
| 21 | Feleke DG et al. (60) | Y | Y | N | Y | N | Y | N | Y | 5/8 | Included |
| 22 | Megersa S et al. (100) | Y | Y | N | Y | N | Y | N | Y | 5/8 | Included |
| 23 | Beyene SG et al. (101) | Y | Y | N | Y | N | Y | N | Y | 5/8 | Included |
| 24 | Kasee LF et al. (102) | Y | Y | N | Y | N | N | N | Y | 4/8 | Included |
| 25 | Gashaw TA et al. (26) | Y | Y | N | Y | N | Y | N | Y | 5/8 | Included |
| 26 | Bitew BD et al. (34) | Y | Y | N | Y | N | Y | N | Y | 5/8 | Included |
| 27 | Getachew F et al. (58) | Y | Y | N | Y | N | Y | N | Y | 5/8 | Included |
| 28 | Zedie FB et al. (31) | Y | Y | N | Y | N | Y | N | Y | 5/8 | Included |
| 29 | Chomissa AR et al. (23) | Y | Y | N | Y | N | Y | N | Y | 5/8 | Included |
| 30 | Arba A et al. (103) | Y | Y | N | Y | N | N | N | Y | 4/8 | Included |
| 31 | Amamo DD et al. (50) | Y | Y | N | Y | N | Y | N | Y | 5/8 | Included |
| 32 | Zegeye Z (57) | Y | Y | N | Y | N | Y | N | Y | 5/8 | Included |
| 34 | Mengistu KD (49) | Y | Y | N | Y | N | Y | N | Y | 5/8 | Included |
| 40 | Angasu K et al. (9) | Y | Y | N | Y | N | Y | N | Y | 5/8 | Included |
| 41 | Mitiku HD (16) | Y | Y | N | Y | N | Y | N | Y | 5/8 | Included |
| 44 | Kassie G (104) | Y | Y | N | Y | N | Y | N | Y | 5/8 | Included |
| 46 | Alemayehu B et al. (24) | Y | Y | N | Y | N | Y | N | Y | 5/8 | Included |
| 47 | Natnael T et al. (12) | Y | Y | N | Y | N | Y | N | Y | 5/8 | Included |
| 48 | Bekele D et al. (25) | Y | Y | N | Y | N | Y | N | Y | 5/8 | Included |
| 49 | Fenta A et al. (45) | Y | Y | N | Y | N | Y | N | Y | 5/8 | Included |
| 50 | Alemayehu K et al. (11) | Y | Y | N | Y | N | Y | N | Y | 5/8 | Included |
| 51 | Shine S et al. (21) | Y | Y | N | Y | N | Y | N | Y | 5/8 | Included |
| 52 | Solomon ET et al. (46) | Y | Y | N | Y | N | Y | N | Y | 5/8 | Included |
| 53 | Tafere Y et al. (29) | Y | Y | N | Y | N | Y | N | Y | 5/8 | Included |
| 54 | Mulu E et al. (35) | Y | Y | N | Y | N | Y | N | Y | 5/8 | Included |

Y: Yes; N: No; NA: Unclear/Not applicable; Quality: low (0 to 2), moderate (3 or 4), and high (total score of 5 or higher)

1. Were the criteria for inclusion in the sample clearly defined?

2. Were the study subjects and the setting described in detail?

3. Was the exposure measured in a valid and reliable way?

4. Were objective, standard criteria used for measurement of the condition?

5. Were confounding factors identified?

6. Were strategies to deal with confounding factors stated?

7. Were the outcomes measured in a valid and reliable way?

8. Was appropriate statistical analysis used?

**Table 2: Critical appraisal checklist for observational studies (Joanna Briggs Institute) in the systematic review according to the 10 items (*for case-control studies*)**

|  | **Author** | **1** | **2** | **3** | **4** | **5** | **6** | **7** | **8** | **9** | **10** | **Final score** | **Overall Appraisal** |
| --- | --- | --- | --- | --- | --- | --- | --- | --- | --- | --- | --- | --- | --- |
| 1 | Brhanu H et al. (14) | Y | Y | Y | Y | Y | N | Y | N | NA | Y | 7/10 | Included |
| 2 | Derseh BT et al. (30) | Y | Y | Y | Y | Y | N | Y | N | NA | Y | 7/10 | Included |
| 3 | Delelegn MW et al. (41) | Y | Y | Y | Y | Y | N | Y | N | NA | Y | 7/10 | Included |
| 4 | Mosisa D et al. (10) | Y | Y | Y | Y | Y | N | Y | N | NA | Y | 7/10 | Included |
| 5 | Baye A et al. (19) | Y | Y | Y | Y | Y | Y | Y | N | NA | Y | 8/10 | Included |
| 6 | Soboksa NE et al. (32) | Y | Y | Y | Y | Y | Y | Y | N | NA | Y | 8/10 | Included |
| 7 | Girma M et al. (15) | Y | Y | Y | Y | Y | N | Y | N | NA | Y | 7/10 | Included |
| 8 | Brhanemeskel H (53) | Y | Y | Y | Y | Y | N | Y | N | NA | Y | 7/10 | Included |
| 9 | Getachew B et al. (20) | Y | Y | Y | Y | Y | N | Y | N | NA | Y | 7/10 | Included |

Y: Yes; N: No; NA: Unclear/Not applicable; Quality: low (0 to 2), moderate (3 or 4), and high (total score of 5 or higher)

1. Were the groups comparable other than the presence of disease in cases or the absence of disease in controls?

2. Were cases and controls matched appropriately?

3. Were the same criteria used for identification of cases and controls?

4. Was exposure measured in a standard, valid and reliable way?

5. Was exposure measured in the same way for cases and controls?

6. Were confounding factors identified?

7. Were strategies to deal with confounding factors stated?

8. Were outcomes assessed in a standard, valid and reliable way for cases and controls?

9. Was the exposure period of interest long enough to be meaningful?

10. Was appropriate statistical analysis used?
