# Supplementary material for: Burden of Childhood Diarrhea and Its Associated Factors in Ethiopia: A Review of Observational Studies
Source: Int J Public Health. 2024 Jun 5;69:1606399. doi: 10.3389/ijph.2024.1606399 (PMC11188320; doi:10.3389/ijph.2024.1606399)
Supplement: Supplementary file 5 [file DataSheet5.docx]

**Supplementary file 5: Sensitivity analysis using a random-effects model**
