# Supplementary material for: Burden of Childhood Diarrhea and Its Associated Factors in Ethiopia: A Review of Observational Studies
Source: Int J Public Health. 2024 Jun 5;69:1606399. doi: 10.3389/ijph.2024.1606399 (PMC11188320; doi:10.3389/ijph.2024.1606399)
Supplement: Supplementary file 7 [file DataSheet2.docx]

**Supplementary file 2**

**Example of searching strategy for PubMed**

1. diarrhea [MeSH Terms] OR Diarrhea [Text Word] OR Diarrhoea [All Filed] OR Dysentery [All Filed] OR Liquid faeces [All Filed]
2. Factor* OR determinant* OR correlate* OR cause*
3. child [MeSH Terms] OR child [Text Word] OR "infant"[MeSH Terms] OR infant[Text Word] OR "child, preschool"[MeSH Terms] OR "under-five children" [Text Word] OR "children" [Text Word]
4. "ethiopia"[MeSH Terms] OR Ethiopia[Text Word]
5. **(**diarrhea [MeSH Terms] OR Diarrhea [Text Word] OR Diarrhoea [All Filed] OR Dysentery [All Filed] OR Liquid faeces [All Filed]) OR (Factor* OR determinant* OR correlate* OR cause*)
6. **(**diarrhea [MeSH Terms] OR Diarrhea [Text Word] OR Diarrhoea [All Filed] OR Dysentery [All Filed] OR Liquid faeces [All Filed]) OR (Factor* OR determinant* OR correlate* OR cause*) AND (child [MeSH Terms] OR child [Text Word] OR "infant"[MeSH Terms] OR infant[Text Word] OR "child, preschool"[MeSH Terms] OR "under-five children" [Text Word] OR "children" [Text Word])
7. **(**diarrhea [MeSH Terms] OR Diarrhea [Text Word] OR Diarrhoea [All Filed] OR Dysentery [All Filed] OR Liquid faeces [All Filed]) OR (Factor* OR determinant* OR correlate* OR cause*) AND (child [MeSH Terms] OR child [Text Word] OR "infant"[MeSH Terms] OR infant[Text Word] OR "child, preschool"[MeSH Terms] OR "under-five children" [Text Word] OR "children" [Text Word]) AND "ethiopia"[MeSH Terms] OR Ethiopia[Text Word]
